# *hipBA* toxin-antitoxin systems mediate persistence in *Caulobacter crescentus*

**DOI:** 10.1038/s41598-020-59283-x

**Published:** 2020-02-18

**Authors:** Charlie Y. Huang, Carlos Gonzalez-Lopez, Céline Henry, Ivan Mijakovic, Kathleen R. Ryan

**Affiliations:** 1 Department of Plant & Microbial Biology, University of California, Berkeley, USA; 2 Université Paris-Saclay, AgroParisTech, Micalis Institute, PAPPSO, INRAE, 78350 Jouy-en-Josas, France; 3 Division of Systems and Synthetic Biology, Department of Chemical and Biological Engineering, Chalmers University of Technology, Gothenburg, Sweden; 4 Novo Nordisk Foundation Center for Biosustainability, Technical University of Denmark, Kongens Lyngby, Denmark

**Keywords:** Bacterial toxins, Bacterial genes

## Abstract

Antibiotic persistence is a transient phenotypic state during which a bacterium can withstand otherwise lethal antibiotic exposure or environmental stresses. In *Escherichia coli*, persistence is promoted by the HipBA toxin-antitoxin system. The HipA toxin functions as a serine/threonine kinase that inhibits cell growth, while the HipB antitoxin neutralizes the toxin. *E. coli* HipA inactivates the glutamyl-tRNA synthetase GltX, which inhibits translation and triggers the highly conserved stringent response. Although *hipBA* operons are widespread in bacterial genomes, it is unknown if this mechanism is conserved in other species. Here we describe the functions of three *hipBA* modules in the alpha-proteobacterium *Caulobacter crescentus*. The HipA toxins have different effects on growth and macromolecular syntheses, and they phosphorylate distinct substrates. HipA_1_ and HipA_2_ contribute to antibiotic persistence during stationary phase by phosphorylating the aminoacyl-tRNA synthetases GltX and TrpS. The stringent response regulator SpoT is required for HipA-mediated antibiotic persistence, but persister cells can form in the absence of all *hipBA* operons or *spoT*, indicating that multiple pathways lead to persister cell formation in *C. crescentus*.

## Introduction

Toxin-antitoxin (TA) modules are small operons that encode a toxic protein and a corresponding antitoxin^1^. In Type II TA systems, the antitoxin is a protein that binds and neutralizes the toxin^2^. When free of inhibition, the toxin acts on various targets to inhibit cell growth or cause death^3^. While the toxin is long-lived, the antitoxin is labile and degraded by proteases. Therefore, the antitoxin must be continually produced to keep the toxin in check^4^. Both the antitoxin alone and the toxin-antitoxin complex can repress their own transcription; thus, as the antitoxin is degraded, the repression is relieved and the operon is transcribed to replenish the antitoxin supply^5,6^.

TA modules were initially found to promote plasmid maintenance via post-segregational killing of cells that did not inherit a plasmid^7^. Subsequently, TA modules were found to be highly abundant in the chromosomes of almost all free-living bacteria, raising questions about additional biological functions^2,8^. TA systems can provide stability to mobile genetic elements in bacterial chromosomes, but a growing body of work indicates that TA modules are involved in additional processes including biofilm formation, phage resistance, stress responses, and antibiotic persistence^9–13^.

Antibiotic persistence plays an important role in chronic infections and facilitates the evolution of antibiotic resistance^14^. In contrast to resistance, in which a heritable genetic change renders an entire bacterial population able to grow in the presence of an antibiotic, persister cells are genetically identical to their susceptible relatives and they tolerate antibiotics and other stresses by entering a transient, non-replicating state^15,16^. Persister cells can resume normal growth once the stressor is removed, but they are once again sensitive to the stress^16^. Persistence is viewed as a bet-hedging strategy against unexpected environmental threats; the dormant cells temporarily sacrifice replication in exchange for stress tolerance^17,18^.

TA systems were first implicated in antibiotic persistence when a screen for *E. coli* mutants with increased levels of persister cell formation retrieved the gain-of-function *hipA*7 allele^19^. Named for *h*igh *i*ncidence of *p*ersistence, the *hipBA* module encodes two proteins, the HipB antitoxin and the HipA toxin, which functions as a serine/threonine kinase^20–22^. A G22S amino acid substitution in HipA7 is thought to increase the likelihood of HipA activity by interfering with HipA dimerization within HipA/HipB/operator complexes at the *hipBA* promoter^23,24^. Mutations in *hipA* are reported to arise in half of *E. coli* clinical isolates associated with chronic urinary tract infections, indicating a need for further studies to understand how HipA toxins promote antibiotic persistence^24^.

To date, the HipBA system has only been mechanistically studied in *E. coli*, where free HipA phosphorylates and inactivates the glutamyl-tRNA synthetase GltX^25^. The accumulation of uncharged tRNAs is detected by the ribosome-associated protein RelA, which synthesizes the alarmone (p)ppGpp to signal a state of amino acid starvation, known as the stringent response^26,27^. (p)ppGpp binding to target proteins, such as RNA polymerase and primase, reprograms transcription, downregulates macromolecular syntheses, and promotes dormancy^28^. However, other substrates of *E. coli* HipA have recently been identified, and additional phosphorylation events may stimulate persister formation^26^.

*hipBA* operons are present in numerous, phylogenetically distinct bacterial genomes, yet it is unknown if all HipBA modules influence persistence, or if all HipA toxins phosphorylate the same substrates. To address these questions, we are studying HipBA systems in *Caulobacter crescentus* NA1000, a Gram-negative alpha-proteobacterium that lives in nutrient-limited freshwater environments and maintains three *hipBA* operons in its chromosome^29,30^. Here we report that all three *hipBA* operons encode active TA systems, and that two of them are responsible, via the stringent response, for the majority of antibiotic persistence during stationary phase. The three HipA toxins phosphorylate distinct sets of substrates, of which specific aminoacyl-tRNA synthetases are critical targets for the development of antibiotic persistence. Importantly, persister cells are still observed after elimination of all three *hipBA* operons or the stringent response regulator *spoT*, indicating that there are multiple pathways leading to antibiotic persistence in *C. crescentus*.

## Results

### All three *hipBA* modules are *bona fide* toxin-antitoxin systems

**Figure 1:**
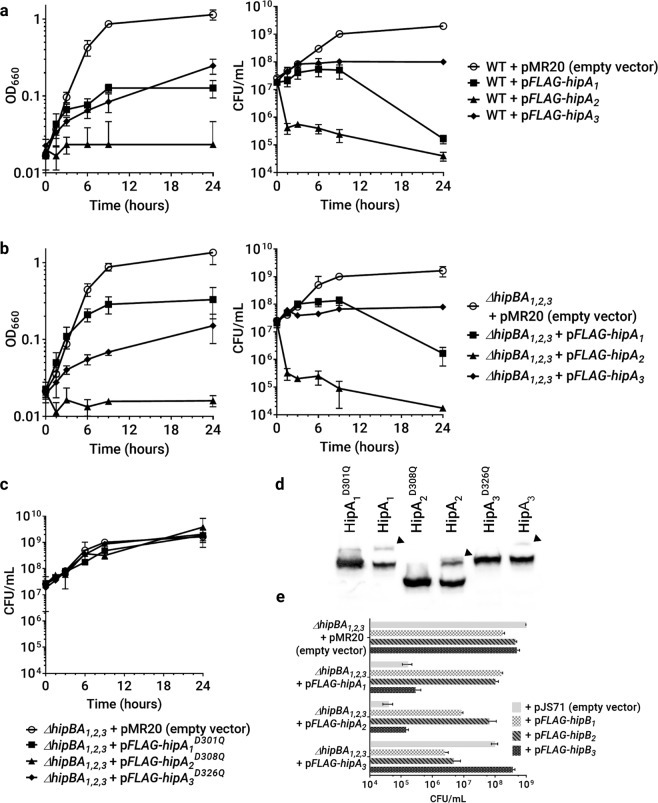
All NA1000 *hipBA* operons encode functional TA modules. Growth curves of NA1000 (**a**) or *ΔhipBA*_1,2,3_ (**b**,**c**) cells expressing the indicated HipA toxins in exponential phase. HipA proteins were induced at time 0 by the addition of IPTG and theophylline. (**d)** The indicated HipA toxins were expressed in *ΔhipBA*_1,2,3_ cells in exponential phase for 1.5 hours beginning at OD_660_ = 0.02. Whole-cell lysates were analyzed by Phos-tag mobility shift and Western blotting with anti-FLAG antibodies. Arrowheads indicate phosphorylated species with reduced mobility. A cropped gel image is shown here, and the complete gel is shown in Supplementary Fig. S9. **(e**) The indicated HipA toxins were expressed from low-copy plasmids, and the indicated HipB antitoxins were expressed from high-copy plasmids, each under control of the RiboA promoter. Exponential phase cultures in PYE were subcultured to OD_660_ = 0.02 and supplemented with IPTG and theophylline to induce protein expression. After 18 hours, samples were collected for CFU enumeration. Values reported are the mean and standard deviation from three independent biological replicates.

A blastp search of the NA1000 genome for proteins similar to *E. coli* HipA revealed three predicted *hipBA* operons: *hipBA*_1_, *CCNA_*00481*-*2; *hipBA*_2_, *CCNA_*02822*-*1; and *hipBA*_3_, *CCNA_*02859*-*8. When ectopically expressed under control of an inducible riboA promoter on a low-copy plasmid, each HipA toxin inhibited the accumulation of cell mass in exponential-phase NA1000 cultures, albeit to different degrees (Fig. 1a, left panel)^31^. When colony-forming units/ml were enumerated, HipA_1_ and HipA_2_ significantly reduced cell viability, while HipA_3_ caused cell viability to plateau (Fig. 1a, right panel). *C. crescentus* cultures expressing HipA_3_ continue to increase in optical density (Fig. 1a, left panel) without a concomitant increase in viable cell number because a portion of the cells become elongated (Supplementary Fig. S1).

While no bacterium with multiple HipA toxins has been studied, co-activation of distinct TA systems within the same organism has been reported, and we could not discount the possibility of crosstalk between *hipBA* modules^32,33^. To assess the effect of each HipA toxin without the possibility of crosstalk, we expressed each HipA from a low-copy plasmid in a strain with all three *hipBA* operons deleted from the chromosome (*ΔhipBA*_1,2,3_). Each HipA toxin independently inhibited growth in this strain (Fig. 1b**)**. HipA_2_ or HipA_3_ caused slightly greater losses of viability in *ΔhipBA*_1,2,3_ than in NA1000, likely due to the absence of chromosomally encoded HipB antitoxins that would mitigate the effects of ectopic HipA expression. In contrast, HipA_1_ was somewhat less toxic when expressed in the *ΔhipBA*_1,2,3_ strain than in NA1000, suggesting that its deleterious effects are enhanced in wild-type cells by coactivation of another HipA (see below).

All three HipA toxins maintain the conserved active site residues of serine/threonine kinases (Supplementary Fig. S2)^34^. We generated an aspartate-to-glutamine (D-Q) substitution in the active site of each HipA, modeled on a corresponding substitution used to create a kinase-dead *E. coli* HipA protein^35^. Each HipA D-Q variant was ectopically expressed in the *ΔhipBA*_1,2,3_ strain to determine if kinase activity is necessary cause a plateau or reduction in viability (Fig. 1c). Although each kinase-dead HipA protein was expressed (Fig. 1d), we observed no growth inhibition, indicating that the toxic effects of the wild-type HipA proteins are mediated by phosphorylation of downstream targets.

Once released from antitoxin inhibition, *E. coli* HipA eventually autophosphorylates on a serine residue, which structurally blocks its kinase activity^35^. In persister cells generated due to HipA activity, autophosphorylation is likely important for the resumption of normal growth, also known as persister resuscitation^35^. To confirm the kinase activity of each HipA protein, we examined autophosphorylation using Phos-tag mobility shift assays and Western blotting. When expressed in the *ΔhipBA*_1,2,3_ strain, we observed retardation of a portion of each wild-type HipA protein, indicative of autophosphorylation (Fig. 1d). We observed no mobility shift when kinase-dead HipA_2_ or HipA_3_ variants were expressed. A small amount of apparently retarded HipA_1_-D301Q protein could either be part of a smear of unshifted protein or could represent a small amount of autophosphorylated protein (Fig. 1d). Further studies are needed to clarify the effects of the D301Q substitution in HipA_1_. However, since ectopic expression of HipA_1_-D301Q did not reduce *Caulobacter* viability (Fig. 1c), this amino acid substitution impairs its toxicity in some way.

We expected each HipA toxin to be counteracted by binding to the HipB antitoxin encoded in its operon. Promiscuity between toxin and antitoxin partners is rare, even in bacteria harboring multiple paralogous toxins, but crosstalk in HipA-HipB interactions has never been investigated^36,37^. To determine the cognate HipB antitoxin(s) for each HipA toxin, we coexpressed all combinations of HipA and HipB in a *ΔhipBA*_1,2,3_ background (Fig. 1e). As expected, the growth arrest caused by ectopically expressing each HipA was mitigated by coexpressing the HipB in its own operon. Interestingly, the toxicity of HipA_1_ or HipA_2_ was also counteracted by coexpression of HipB_2_ or HipB_1_, respectively, indicating that the *hipBA*_1_ and *hipBA*_2_ modules can influence each other’s phenotypic effects. Thus, overexpression of HipA_1_ in a wild-type strain, as in Fig. 1a, could coactivate the HipA_2_ kinase by binding to and titrating away the cellular supply of HipB_2_.

### HipA toxins in NA1000 do not require the stringent response to arrest growth and are not sufficient for population-wide activation of the stringent response

*E. coli* HipA is significantly less toxic in a strain that lacks RelA, and HipA has been proposed to rely on the stringent response to inhibit DNA replication and transcription^27^. However, it is not known if interaction with the stringent response is a universal feature of HipA activity. The stringent response in *C. crescentus* relies on a single (p)ppGpp synthetase/hydrolase SpoT^38^. SpoT associates with the translating ribosome and responds to amino acid limitation, but only in combination with a separate cue indicating either carbon or nitrogen starvation^39^. Thus, the accumulation of uncharged tRNAs, such as those produced by HipA-mediated phosphorylation of GltX, would be insufficient on its own to activate the *C. crescentus* stringent response. However, *C. crescentus* HipA_1_ or HipA_2_ could phosphorylate additional substrates which synergize with inactive tRNA synthetases to trigger the stringent response.

**Figure 2:**
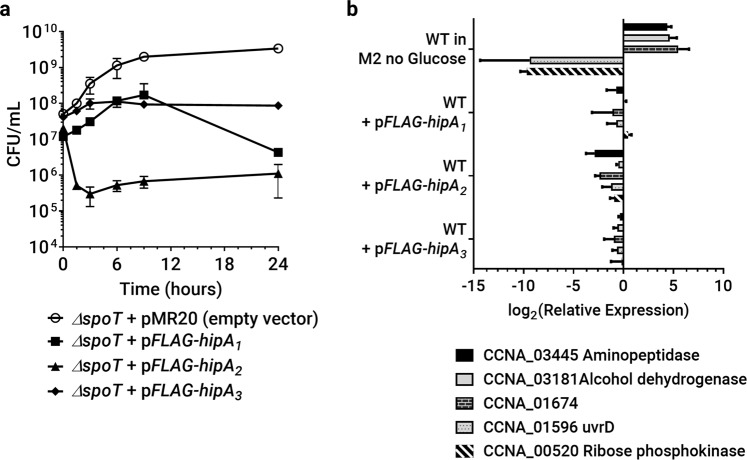
The stringent response is not required for HipA-mediated growth inhibition, and no HipA is sufficient to activate the stringent response in exponential phase cultures. (**a**) Growth curves of *ΔspoT* ectopically expressing each indicated HipA toxin performed as in Fig. 1a. (**b)** The indicated strains in exponential phase in M2G were subcultured to OD_660_ = 0.02 in M2G supplemented with IPTG and theophylline to express HipA toxins. As a positive control, the wild-type NA1000 was subcultured in M2 medium lacking the carbon source to induce the stringent response. RNA was harvested after 3 hours of HipA expression or carbon starvation, and the indicated genes were subjected to RT-PCR analysis as described in Methods. Expression relative to an unstarved M2G control is reported, and values are the mean and standard deviation of three biological replicates, each used in two technical replicates.

To investigate the relationship between *Caulobacter* HipAs and the stringent response, we first asked if SpoT was required for HipA toxicity in NA1000. Expression of each HipA toxin had the same effect upon cell viability in a *ΔspoT* mutant as in NA1000 (Figs. 1a and 2a). Thus, HipA-induced growth inhibition or reduction in viability is independent of the stringent response. To determine if the stringent response is activated by ectopic HipA expression, we measured the transcription of three genes that are highly upregulated and two genes that are significantly downregulated during the stringent response^39^. In contrast to incubation in minimal medium lacking a carbon source, the expression of individual HipA toxins did not produce the transcriptional changes characteristic of stringent response activation (Fig. 2b**)**. However, RT-PCR measures bulk transcriptional changes occurring across the entire cell population, and stringent response activation in a small fraction of the cells (e.g., 10^−6^ to 10^−3^) would not be detected by this assay.

HipA toxins did produce some transcriptional changes as compared to unstarved controls, but these were smaller in magnitude and sometimes in the opposite direction from those associated with the stringent response. We hypothesize that these smaller transcriptional changes are downstream consequences of HipA expression on cell growth, cell viability, and/or protein synthesis (see below). Taken together, these experiments show that the HipA toxins do not require the stringent response to inhibit cell growth or viability, and their expression is not sufficient to activate the stringent response population-wide in *C. crescentus* during exponential growth.

### HipAs inhibit protein synthesis

**Figure 3:**
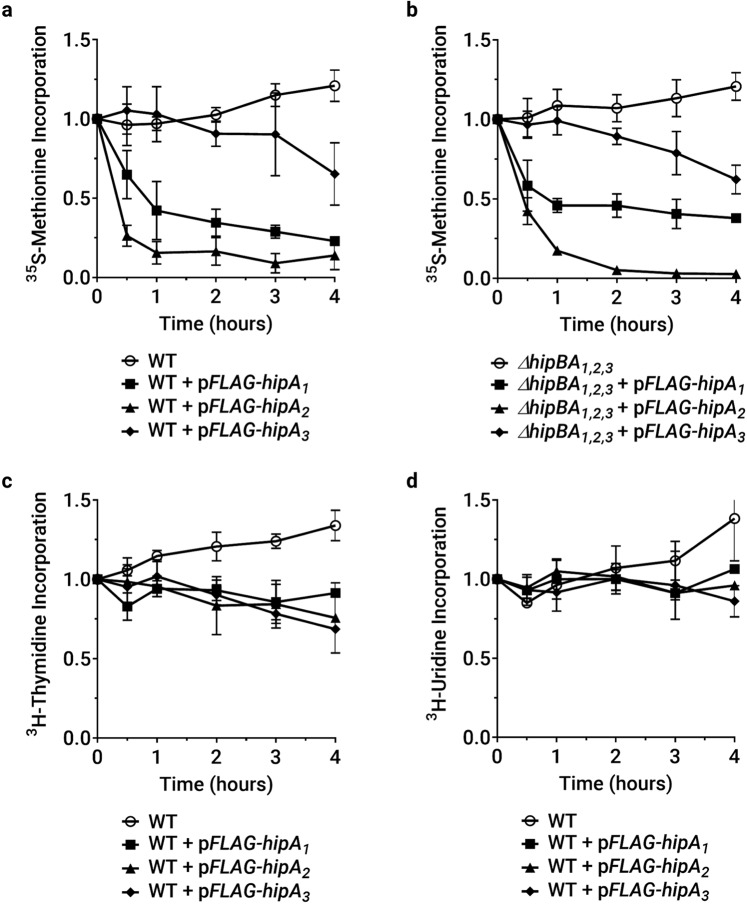
Ectopic expression of HipA toxins inhibits protein synthesis, but not DNA replication or transcription. Assays were conducted in M2G medium to monitor the incorporation of radiolabeled monomers into protein, DNA or RNA. Rates of incorporation are expressed relative to the incorporation of the culture at time zero, before toxin induction. Values reported are the mean and standard deviation of three biological replicates. (**a**,**b**) Relative ^35^S-methionine incorporation when HipA proteins are ectopically expressed in wild-type or *ΔhipBA*_1,2,3_ strain backgrounds. (**c**) Relative ^3^H-thymidine incorporation when HipA is ectopically expressed in NA1000. (**d**) Relative ^3^H-uridine incorporation when HipA is ectopically expressed in NA1000.

The best-understood effects of HipA are upon protein synthesis, but *E. coli* HipA has also been reported to inhibit DNA replication and transcription^25,40^. To identify macromolecular syntheses affected by *C. crescentus* HipA toxins, we measured the bulk incorporation of ^35^S-methionine, ^3^H-thymidine, or ^3^H-uridine following induction of each HipA. ^35^S-methionine incorporation is rapidly reduced to 20% or less of the control level by HipA_2_ expression, and to 50% or less of the control level by HipA_1_, but is reduced more mildly over several hours in cultures expressing HipA_3_ (Fig. 3a,b). Because the bulk biochemical incorporation of ^35^S-methionine is reduced by at least half in cultures expressing HipA_1_ or HipA_2_, we infer that protein synthesis is inhibited population-wide, or in the majority of cells. HipA_2_ inhibits translation slightly more strongly in the *ΔhipBA*_1,2,3_ background, possibly because this strain contains no chromosomally produced HipB_2_ or HipB_1_ toxin to restrain its activity. In contrast, HipA_1_ inhibits protein synthesis more weakly in the *ΔhipBA*_1,2,3_ strain, consistent with its weaker effects upon biomass accumulation and viability in *ΔhipBA*_1,2,3_ cells **(**Fig. 1a,b), and consistent with the hypothesis that HipA_1_ can coactivate HipA_2_ in wild-type cells by titrating away the cellular supply of HipB_2_. ^3^H-thymidine incorporation is mildly reduced after several hours when any HipA is expressed (Fig. 3c); however, this may be due to indirect effects of growth arrest. ^3^H-uridine incorporation was unchanged when any HipA was expressed, indicating that RNA synthesis is not targeted (Fig. 3d). Thus, HipA_1_ and HipA_2_ sharply and quickly inhibit the translation, while no *C. crescentus* HipA toxin strongly inhibits DNA or RNA synthesis.

### HipA_1_ and HipA_2_ phosphorylate tRNA synthetases

**Figure 4:**
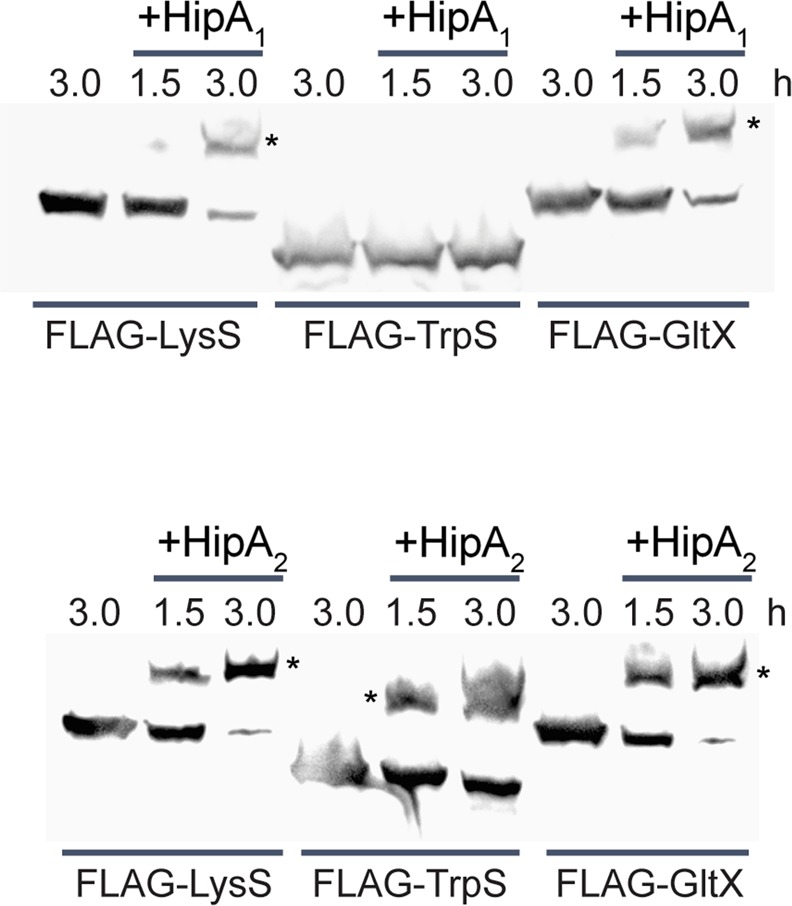
Ectopic HipA_1_ or HipA_2_ induction leads to the phosphorylation of distinct aminoacyl-tRNA synthetases. Strains expressed the indicated HipA toxins from low-copy plasmids and the indicated aminoacyl-tRNA synthetases from high-copy plasmids, each under control of the RiboA promoter. Exponential phase PYE cultures were subcultured to OD_660_ = 0.02 and supplemented with IPTG and theophylline to induce expression of tRNA synthetases alone, or HipA toxins and aminoacyl-tRNA synthetases together. Samples were collected at the indicated times after protein induction and processed as described in Methods for Phos-tag mobility shift analysis and Western blotting with anti-FLAG antibodies. Asterisks mark phosphorylated protein species with reduced mobility. Cropped images are shown here, and full images of blots are found in Supplementary Fig. S10.

From an initial phosphoproteomics analysis of unstressed NA1000 during exponential growth (Supplementary Table S1), we noted that the aminoacyl-tRNA synthetases GltX, LysS, and TrpS were phosphorylated on serine residues. No kinase was previously known to phosphorylate these proteins, and the HipA and Doc toxins are the only predicted serine/threonine kinases encoded in the NA1000 genome. By analogy with *E. coli* HipA, and based on their inhibition of protein synthesis, we hypothesized that HipA_1_ and/or HipA_2_ phosphorylate GltX, TrpS, and LysS. We used Phos-tag mobility shift assays to examine the phosphorylation of aminoacyl-tRNA synthetases in *ΔhipBA*_1,2,3_ cells expressing individual HipA toxins. HipA_2_ expression caused retardation of LysS, TrpS and GltX, while HipA_1_ expression slowed the migration of LysS and GltX (Fig. 4). Consistent with its much weaker effect on protein synthesis, HipA_3_ did not affect the migration of LysS or TrpS and only caused the retardation of a small fraction of GltX after an extended period of toxin expression (Supplementary Fig. S3). Although it is formally possible that HipA_1_ and/or HipA_2_ activate an unknown kinase that phosphorylates these targets, the simplest interpretation is that HipA_1_ and HipA_2_ directly phosphorylate overlapping sets of aminoacyl-tRNA synthetases, which inhibits translation.

### *hipBA*_1_ and *hipBA*_2_ contribute to persistence in *C. crescentus* via phosphorylation of TrpS and GltX

Antibiotic persistence has not previously been reported in *C. crescentus*. We used Minimum Duration for Killing (MDK) assays to measure bacterial survival over time after exposure to saturating antibiotic concentrations (10–500 times the minimal inhibitory concentration)^41^. When plotted, cultures that contain a sensitive population and a persister fraction generate a biphasic killing curve that reflects the different survival rates of the two populations (Supplementary Fig. S4). An increase in persister fraction shifts the second phase of the curve upwards, while a decrease moves the second phase downwards^42^. The biphasic curve can be used to estimate the frequency of persister cells and describe the survival rate over time of each population. While much of the work on persistence and HipA employs beta-lactam antibiotics to kill the susceptible population, *C. crescentus* NA1000 expresses an active beta-lactamase enzyme Bla^43^. We observed persistence toward carbenicillin in a *Δbla* strain (Supplementary Fig. S5), but we focus on other antibiotics to dissect the contributions of the *hipBA* modules to persistence in *C. crescentus*.

**Figure 5:**
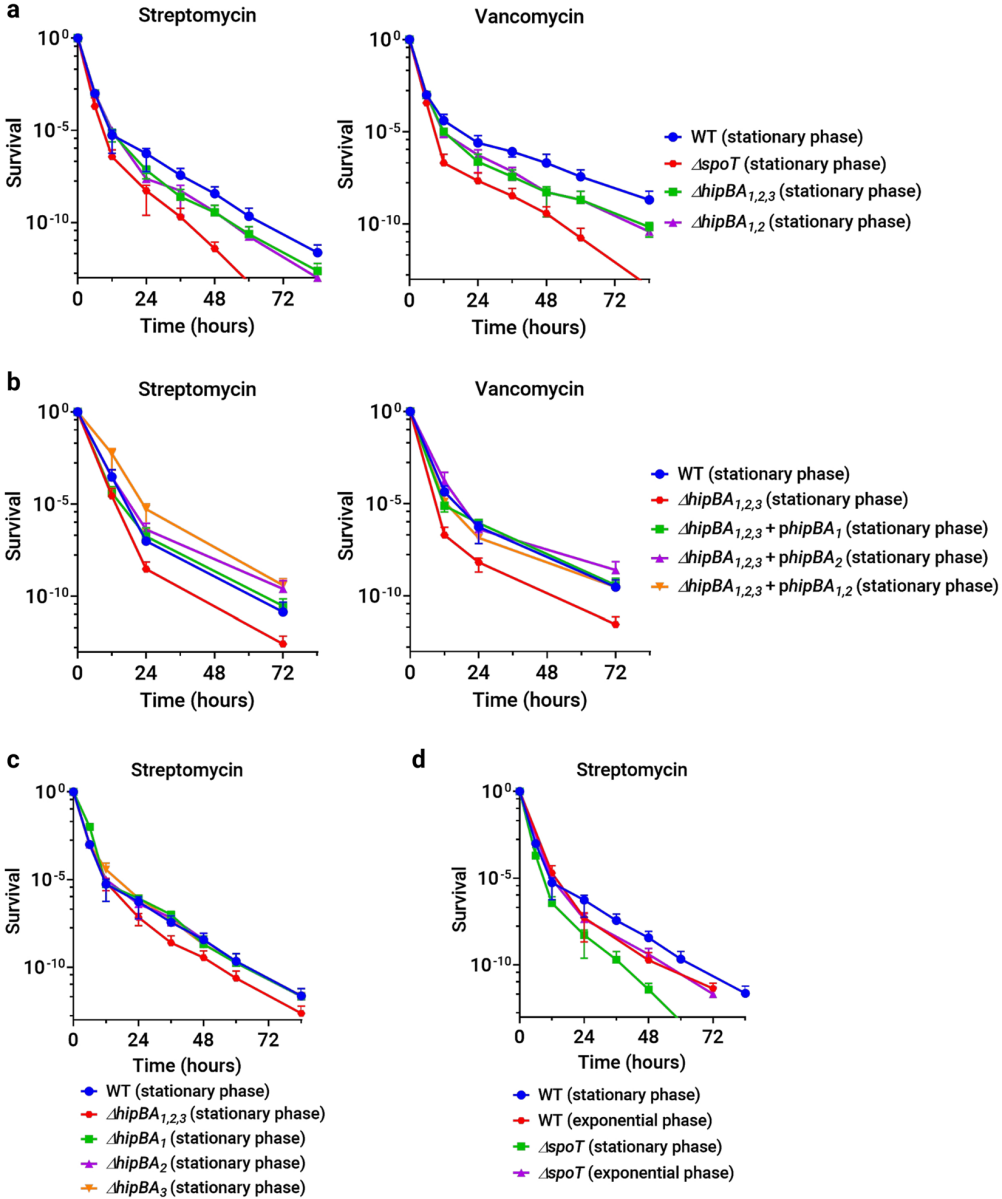
HipBA_1_, HipBA_2_, and SpoT contribute to persister cell formation in stationary phase *C. crescentus* cultures. (**a**) Survival of *ΔhipBA*_1,2,3_, *ΔhipBA*_1,2_, and *ΔspoT* strains grown to stationary phase in PYE medium and treated with streptomycin or vancomycin. (**b**) Survival of a *ΔhipBA*_1,2,3_ strain with plasmids bearing wild-type *hipBA*_1_ and/or *hipBA*_2_ operons grown and tested under the same conditions as in (**a)**. (**c**) Survival of single *hipBA* operon knockout strains grown to stationary phase and treated with streptomycin as in (**a)**. (**d**) Comparison of the survival of exponential and stationary phase cultures of NA1000 and *ΔspoT* strains treated with streptomycin.

We selected an aminoglycoside, streptomycin, and a cell wall synthesis inhibitor, vancomycin, for their rapid bactericidal activity against *C. crescentus*. We observed a biphasic killing curve in exponential phase cultures (Fig. 5d); however, the lower frequency of persistence and the lower cell density during exponential growth make measurements challenging, as the second phase of the killing curve quickly moves below the detection limit of our assay. These results are consistent with reports of other bacteria, where persisters are rarer during exponential growth than during stationary phase^15,44^. Consequently, we focused on persistence during stationary phase. In NA1000 cultures that had reached full cell density (OD_660_~1.0, after 24 hours of growth from OD_660_ = 0.02), we observed biphasic killing when saturating antibiotic concentrations were applied. The wild-type persister fraction was estimated to be ~10^−4^–10^−5^ in stationary phase cultures grown in PYE medium and was consistent across carbenicillin, streptomycin and vancomycin treatments (Supplementary Figs. S5 and 5a,b).

To determine if one or more *C. crescentus hipBA* modules contribute to persistence, we first measured the persister fraction in a strain lacking all *hipBA* operons (Fig. 5a). The *ΔhipBA*_1,2,3_ strain showed a significant ~5–10-fold reduction in persister frequency in stationary phase cultures grown in PYE medium when treated with streptomycin or vancomycin. Persisters were not entirely abolished in the *ΔhipBA*_1,2,3_ strain, and the remaining persister fraction had a survival rate similar to that of wild-type persisters. We conclude from these results that at least one *hipBA* module is required for the formation of >80% of *C. crescentus* persister cells in stationary phase, but that persisters which do form in the absence of *hipBA* modules are not impaired in their ability to withstand antibiotic killing.

To dissect the contributions of individual *hipBA* modules, we measured persistence in individual and double *hipBA* knockout strains. Single *hipBA* deletions had no significant effects (Fig. 5c), but we observed the same ~5–10-fold reduction in persister frequency in a *ΔhipBA*_1,2_ strain as in *ΔhipBA*_1,2,3_ (Fig. 5a,b). The reduction in persister frequency in *ΔhipBA*_1,2,3_ cells was complemented by low-copy plasmids bearing *hipBA*_1_ and/or *hipBA*_2_ (Fig. 5b). All strains lacking one or more *hipBA* operons have growth rates very similar to the wild-type strain (Supplementary Fig. S6), so the reduction in persister frequency is not likely to be caused by a pleiotropic fitness defect. We infer that *hipBA*_1_ and *hipBA*_2_ are the primary determinants of persister cell formation in stationary phase, and that there is some level of functional redundancy between these modules. In contrast, the *hipBA*_3_ module appears to play no significant role in antibiotic persistence during stationary phase.

**Figure 6:**
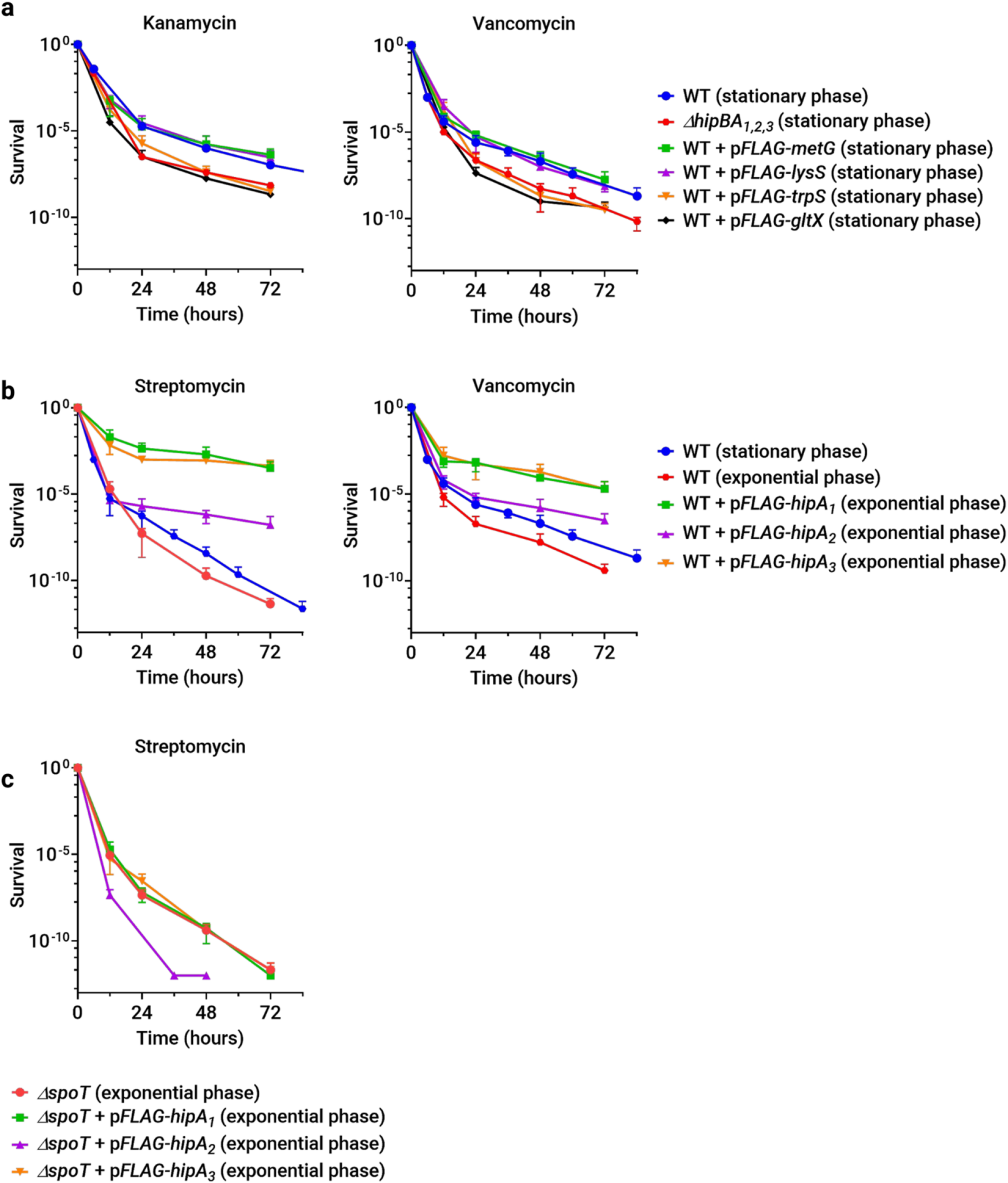
The stationary phase persister fraction can be modulated by expressing HipA toxins or specific tRNA synthetase targets. (**a**) Survival of wild-type strains expressing the indicated tRNA synthetases grown to stationary phase in PYE medium and treated with kanamycin or vancomycin. (**b**) Wild-type strains were induced to express the indicated HipA toxins for three hours in exponential phase before treatment with streptomycin or vancomycin. (**c**) Survival of *ΔspoT* cells expressing the indicated HipA toxins under the conditions described in (**b)** and treated with streptomycin.

Because HipA_1_ and HipA_2_ lead to the phosphorylation of tRNA synthetases, we asked if these targets are important for persister cell formation. Overexpression of GltX or TrpS reduced the persister fraction in stationary phase to the same level as seen in *ΔhipBA*_1,2_ cultures (Fig. 6a). In contrast, strains expressing LysS or the control, methionyl-tRNA synthetase MetG, had persister frequencies indistinguishable from NA1000. Overexpression of GltX or TrpS could reduce the persister fraction either by bolstering tRNA synthetase activity and maintaining active translation, or by titrating HipA_1_ or HipA_2_ away from substrates that are truly critical for persister cell formation. The fact that overexpression of LysS, also a target of HipA_1_ and HipA_2_, was not able to reduce the persister fraction argues against a nonspecific titration effect. These results strongly suggest that GltX and TrpS are critical targets through which HipA_1_ and HipA_2_ mediate persister cell formation, and they demonstrate further that not all phosphorylated substrates are important for HipA-mediated persistence.

### The stringent response is required for the accumulation of persister cells in stationary phase

Although ectopic HipA expression in exponential phase cells did not activate the stringent response and was not required for HipA-induced growth arrest (Fig. 2), the stringent response is important for persister cell formation in some bacteria^45^. We therefore asked if the (p)ppGpp synthase/hydrolase SpoT affects persistence in *C. crescentus*. *ΔspoT* cells divide slightly faster than a wild-type cells, but they lose viability more quickly than wild-type cells during long-term nutrient-limited conditions like stationary phase (Supplementary Fig. S7) or carbon starvation^38,46^. Biphasic killing was observed in stationary phase cultures of a *ΔspoT* strain grown in PYE medium when challenged with streptomycin or vancomycin, indicating that *C. crescentus* can form persisters without the stringent response. During exponential growth, the *ΔspoT* strain had a persister frequency similar to NA1000 (Fig. 5d). However, the fraction of persisters in stationary phase PYE cultures was reduced in the *ΔspoT* mutant by ~5–10 fold, and both the sensitive and persister populations were killed faster than the corresponding wild-type populations. Significantly, the *ΔspoT* strain had similar persister frequencies in exponential and stationary phases, indicating that SpoT is important for the accumulation of persister cells during stationary phase.

The *ΔspoT* and *ΔhipBA*_1,2_ mutants displayed similar reductions in persister frequency specifically during stationary phase. Thus, HipA toxins may mediate persister formation in stationary phase through the stringent response or vice versa. Since deletion of *hipBA* modules or overexpression of specific aminoacyl-tRNA synthetase substrates reduced persister frequencies (Figs. 5a and 6a**)**, we wondered if expressing HipA toxins themselves could increase the persister fraction. To answer this question, we briefly induced expression of each HipA in NA1000, *ΔhipBA*_1,2,3_, or *ΔspoT* cells prior to antibiotic exposure in the persistence assay. Toxins were expressed during exponential phase because protein induction during stationary phase was inconsistent and yielded noisy, unreproducible MDK measurements. To avoid excessive loss of viability from the more toxic HipA proteins, expression was induced for three hours before washing the cells and adding lethal concentrations of antibiotics. Ectopic expression of each HipA increased the persister frequency in NA1000; however, no HipA could increase the persister frequency in a *ΔspoT* background (Fig. 6b,c). We conclude that while the stringent response is not necessary for the HipA toxins to inhibit growth and/or viability population-wide, HipA-induced persister formation in a small fraction of the population does require an intact stringent response.

## Discussion

The *C. crescentus* NA1000 chromosome contains three functional *hipBA* modules, each with a distinct effect on growth, viability, and macromolecular syntheses when ectopically expressed in exponential-phase cultures. HipA_2_ is the most toxic, reducing translation to ≤25% of the wild-type level within one hour and reducing the viable cell count by 10–100-fold within two hours. HipA_1_ also immediately inhibits protein synthesis, but to a lesser degree than HipA_2_, and begins to reduce viability after 12 hours of expression. HipA_3_, which is bacteriostatic, causes a gradual and less severe reduction in translation over four hours. Since our ectopic expression system produces comparable amounts of the three HipA toxins (Fig. 1d), these phenotypes suggest that each toxin has a different level of kinase activity and/or phosphorylates distinct groups of substrates. In agreement with this prediction, we observed that the HipAs have different activity toward selected aminoacyl-tRNA synthetases: HipA_2_ phosphorylates TrpS, GltX and LysS; HipA_1_ phosphorylates LysS and GltX; and HipA_3_ only weakly phosphorylates GltX. These results pave the way for examining kinase-substrate specificity in HipA toxins and suggest that HipA_3_ arrests growth by a mechanism distinct from HipA_1_ and HipA_2_.

Since *C. crescentus* contains three *hipBA* modules, we assessed the possibility of crosstalk by co-expressing non-cognate HipA and HipB proteins. The toxicity of HipA_1_ or HipA_2_ was counteracted by co-expression of either HipB_1_ or HipB_2_, while HipA_3_ could only be inhibited by HipB_3_. Since non-native promoters were used for co-expression, these results indicate crosstalk at the level of protein-protein interaction, where HipB from one module blocks the activity of HipA from another module. In support of this finding, ectopic HipA_1_ expression is somewhat less toxic (Fig. 1a,b) and less capable of inhibiting protein synthesis (Fig. 3a,b) in the *ΔhipBA*_1,2,3_ strain than in a wild-type strain, suggesting that HipA_1_ achieves some of its toxicity by coactivating HipA_2_. Based on examination of the *hipBA*_1_ and *hipBA*_2_ operons, we speculate that they also engage in transcriptional crosstalk. In *E. coli*, HipB alone or HipA/HipB complexes bind inverted repeat sequences with distinct spacing to autoregulate *hipBA* transcription^24,47^. In the promoter regions of *hipBA*_1_ and *hipBA*_2_, we noted a series of identical inverted repeats that occur in intervals consistent with the higher-order structure of *E. coli* HipA/HipB/promoter complexes (Supplementary Fig. S8). Based on these observations, we speculate that cross-regulation may exist in other bacteria harboring multiple HipBA systems.

In contrast to *E. coli*, ectopic expression of the *C. crescentus* HipA toxins during exponential phase is not sufficient to activate the stringent response across the the entire population. This discrepancy is likely due to the different criteria for stringent response activation in the two bacteria. In *E. coli*, RelA synthesizes (p)ppGpp when uncharged tRNAs enter the ribosome, indicative of amino acid starvation^48,49^. In contrast, *C. crescentus* SpoT synthesizes (p)ppGpp only when it senses amino acid starvation in combination with a separate signal of carbon or nitrogen starvation^39^. Thus, to stimulate the (p)ppGpp synthase activity of SpoT in *C. crescentus*, a HipA toxin expressed during exponential growth would need to phosphorylate targets that generate two independent starvation signals, despite the presence of adequate nutrients in the medium. Our phosphorylation and persistence results imply that HipA_1_ and HipA_2_ inhibit the charging of specific tRNAs, supplying the amino acid starvation signal, but they do not by themselves provide a separate signal indicating nitrogen or carbon starvation.

As an organism adapted to aquatic environments with low nutrient levels, *C. crescentus* may not encounter frequent antibiotic stress. However, persister cells are tolerant of a variety of stresses, such as starvation, that *C. crescentus* may face in a rapidly shifting environment^18^. We report the first measurements of antibiotic persistence in *C. crescentus*, in exponential growth and in stationary phase. The persister frequency was consistently ~10^−5^–10^−6^ during exponential growth and ~10^−4^–10^−5^ in stationary phase across all antibiotics tested (carbenicillin, streptomycin and vancomycin) and was similar to the persister frequencies reported in pathogens^15^.

Deletion mutants reveal that *hipBA*_1_, *hipBA*_2_, and *spoT* play significant roles in *C. crescentus* persister cell formation during stationary phase, while *hipBA*_3_ is dispensable. The *ΔspoT* strain does not have a significantly reduced persister frequency compared to wild-type during exponential growth in PYE medium, but it fails to accumulate persister cells in stationary phase, indicating that the stringent response mediates a large (~5–10 fold) increase in persister cells that normally occurs during stationary phase. Both the sensitive and persister sub-populations of the *ΔspoT* strain were killed more rapidly by antibiotics during stationary phase persistence assays, but not during exponential-phase persistence assays. This difference in survival may reflect the reduced ability of *ΔspoT* cells to survive the nutrient-depleted conditions of stationary phase persistence (Supplementary Fig. S7)^38^, rather than a specific reduction in antibiotic tolerance. In contrast, persister cells formed by *C. crescentus* lacking all three *hipBA* operons were killed by antibiotics at the same rate as wild-type persisters (Fig. 5a,c), suggesting that HipBA TA systems primarily affect persister formation. Importantly, persistence in stationary phase is not abolished even when all *hipBA* operons or *spoT* is deleted, indicating that additional mechanisms can support persister formation.

**Figure 7:**
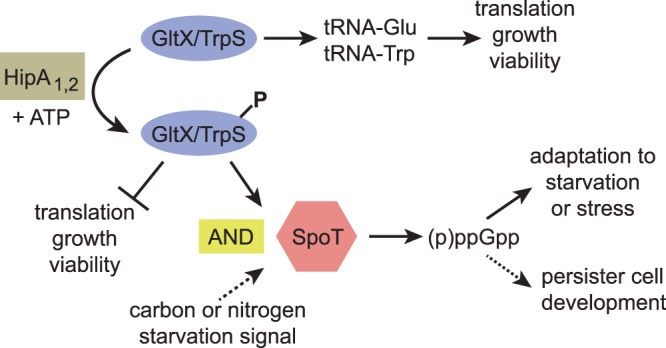
Model of HipA-induced toxicity and persistence. Activated HipA_1_ or HipA_2_ phosphorylates TrpS and/or GltX, preventing the synthesis of charged tRNA-Trp and/or tRNA-Glu. In all cells with activated HipA_1_ or HipA_2_, protein synthesis, growth and viability are inhibited, and a signal of amino acid starvation is delivered to SpoT. However, SpoT is only activated in cells where a second, stochastic signal of carbon or nitrogen starvation is also generated. All cells with activated SpoT become more resistant to stress or starvation, but only a fraction develop into persister cells, possibly due to a second stochastic process downstream of SpoT. Unknown substrates of HipA_1_ or HipA_2_, unpictured, could contribute to growth arrest, viability reduction, or persister cell formation. Solid symbols represent determinant processes, while dotted symbols represent stochastic processes. Arrows indicate production of downstream components or promotion of downstream processes, while bars indicate inhibition of downstream processes.

Ectopic expression of each HipA during exponential growth in a wild-type background increased the persister fraction ~10–100 fold, but only in the presence of an intact *spoT* gene, indicating that all three *C. crescentus* HipA proteins mediate persister formation through the stringent response. These results seemingly contradict the findings that HipA toxins neither induce transcriptional changes associated with the stringent response nor require SpoT to inhibit growth and/or viability. However, unlike our bulk assays of biomass accumulation, transcription, and protein synthesis, the persistence assay measures a cell fate that occurs in only a small fraction of the entire population, at most ~10^−3^ cells in the conditions tested here (Fig. 6b). To reconcile these results, we propose a model in which HipA_1_ or HipA_2_ phosphorylates and inactivates TrpS and/or GltX, inhibiting translation in the entire population and delivering an amino acid starvation signal to SpoT (Fig. 7). However, the stringent response is only activated in a small subset of the population that also receives a signal for nitrogen or carbon starvation. The second signal occurs stochastically, increasing in likelihood as the population enters stationary phase. In *E. coli*, not every cell with an activated stringent response goes on to become a persister. The *E. coli* stringent response is necessary for persister formation, but above some threshold, the level of (p)ppGpp is not predictive of the persistent phenotype^45^. Instead, a stochastic process related to (p)ppGpp-dependent transcriptional reprogramming is thought to drive a sub-population of cells toward the persister fate. By analogy with *E. coli*, *C. crescentus* persister formation in stationary phase may also rely on a second stochastic process downstream of SpoT activation (Fig. 7).

In support of this model, we provide genetic evidence that HipA-mediated phosphorylation of specific aminoacyl-tRNA synthetases contributes to persister formation. Overexpression of GltX or TrpS reduces the persister fraction to a level that phenocopies the *ΔhipBA*_1,2_ strain, but overexpression of another HipA_2_ substrate, LysS, has no effect on persistence. We interpret these results to mean that phosphorylation of GltX and TrpS are critical for SpoT activation and HipA-induced persister formation, whereas LysS phosphorylation is not.

*E. coli* HipA phosphorylates GltX on Ser^239^ within a conserved motif that binds and positions the catalytic ATP molecule^25,26^. Phosphorylation by *E. coli* HipA or substitution of an aspartate residue for Ser^239^ blocked the tRNA charging activity of GltX *in vitro*^25^. In addition, a related toxin, HipT, found specifically in *E. coli* O127, activates the stringent response by phosphorylating TrpS on the analogous residue, Ser^197^,^50^. Our preliminary data suggest that *C. crescentus* TrpS is phosphorylated on the analogous conserved residue, Ser^210^, consistent with HipA_2_ inactivating TrpS. In LysS (encoded by *CCNA_00082*), the analogous serine residue, Ser^322^, was phosphorylated in our phosphoproteome data, yet LysS overexpression did not reduce the fraction of persister cells in stationary phase. These results suggest either that LysS is not inactivated by phosphorylation, or that a second lysyl-tRNA synthetase encoded by *CCNA_00757* provides activity in our conditions. Finally, in our preliminary data, *C. crescentus* GltX is phosphorylated on Ser^2^, rather than Ser^242^, the residue analogous to Ser^239^ in *E. coli* GltX. Ser^2^ is present in a six-amino acid N-terminal peptide not shared by *E. coli* GltX, so we cannot predict how phosphorylation of this residue could inactivate GltX. However, our results raise the interesting possibility that different HipA kinases phosphorylate not only different substrates, but also different sites within shared substrates.

Intriguingly, deletion of *hipBA*_3_ does not reduce the persister fraction in stationary phase, but overexpression of HipA_3_ in exponential phase increases persister formation, dependent upon *spoT*. Based on these results, we propose that HipA_3_ promotes persister formation under environmental conditions that remain to be identified, either by phosphorylating tRNA synthetases that we have not investigated, or by phosphorylating cellular targets that provide a nitrogen or carbon starvation signal to SpoT.

## Methods

### Strains and growth conditions

Strains used in this study are listed in Supplementary Table S2. *Caulobacter* strains are derived from the laboratory strain NA1000. *Caulobacter* strains were grown at 30 °C in rich (PYE) or in minimal (M2G) medium supplemented with antibiotics as required to maintain plasmids^29,51^. *E. coli* was grown in Luria broth at 37 °C, supplemented with antibiotics as appropriate^52,53^

### Plasmid construction

*C. crescentus* NA1000 genomic DNA or purified plasmids were used as template with Q5 High Fidelity DNA Polymerase (New England Biolabs (NEB)) to amplify fragments for cloning. Fragments were isolated from agarose gels using the Zymoclean Gel DNA Recovery kit (Zymo Research). Gibson assembly was performed using NEB Hi-Fi DNA Assembly Master Mix. Plasmid constructs were confirmed by DNA sequencing. Plasmid descriptions are listed in Supplementary Table S3.

### Strain construction

Plasmids for unmarked gene deletions (e.g. *ΔhipBA*_1_) were generated using the pNPTS138 suicide vector, and deletions were made as described using two-step recombination^53,54^. Candidates were screened for the desired deletions by diagnostic PCR using primers indicated in Supplementary Table S4.

### Growth assays

Cells grown to OD_660_ = 0.2–0.5 in PYE were released into PYE at OD_660_ 0.02 and allowed to incubate with shaking at 30 °C. Where indicated, proteins were ectopically expressed from the RiboA promoter by supplementing liquid PYE medium with 1 mM isopropyl thiogalactoside (IPTG) and 1 mM theophylline^31^. Unless otherwise specified, mean and standard deviation of three independent biological replicates are reported.

### DIC microscopy

Cells were immobilized on agarose pads (1% w/v in reverse osmosis-purified water). Microscopy was performed using a Nikon Eclipse 80i microscope with a PlanApo 100×, 1.40 NA objective and a Cascade 512B camera (Roper Scientific). Images were acquired using Metavue software (Universal Imaging).

### RNA extraction and reverse transcription

RNA was extracted using Direct-zol RNA Miniprep Kits from Zymo Research. Purified RNA was reverse transcribed for qPCR using ProtoScript II First-Strand cDNA Transcription kit (NEB) with random hexamers to prime the reaction.

### RT-PCR

RT-PCR was performed on an Applied Biosciences Step One Plus using Luna SYBR Master Mix (NEB). The thermocycling conditions are as follows: initial heating at 95 °C for 20 s, followed by 40 cycles of 95 °C for 3 s, 60 °C for 30 s. *rho* was used as an endogenous control for all experiments. Gene expression relative to control conditions was determined using double delta Ct analysis^55^. Primers used for RT-PCR are listed in Supplementary Table S4.

### Gel detection of phosphoproteins

Samples for phosphoprotein detection were harvested after inducing protein expression for 1.5 or 3 hours from a starting OD_660_ = 0.02 in PYE medium. Cells were washed with PBS containing 1% (v/v) phosphatase inhibitor cocktail (Sigma P5726). Proteins were precipitated using 20% (v/v) trichloroacetic acid overnight at −20 °C. The precipitate was harvested by centrifugation, washed twice with 1 ml ice-cold acetone, and resuspended in 60 μL Tris-HCl pH 8.0 containing 1 mM MnCl_2_ and Laemmli sample buffer. Samples were heated at 95 °C for 5 min, and protein concentration was quantified using Bradford reagent. Samples normalized by protein concentration were loaded into 8% bis-acrylamide gels (Bio-Rad 1610158) containing 5, 10 or 20 μM Phos Binding Reagent (APExBIO F4002) to optimize separation of phosphorylated and non-phosphorylated species by SDS-PAGE. Gels were soaked in Western transfer buffer (25 mM Tris Base, 192 mM glycine, 10% methanol) containing 10 mM EDTA (10 minutes, 3 times) with agitation before a final soak in Western transfer buffer without EDTA. Proteins were transferred to PVDF membranes overnight using Western transfer buffer with 1% SDS on ice. Membranes were probed with anti-FLAG antibodies (1:5000) (Millipore-Sigma F3165) and horseradish peroxidase-conjugated anti-mouse antibodies (1:5000) and analyzed using Western Lightning on a Bio-Rad Gel Doc XR.

### Antibiotic persistence assays

Antibiotic persistence was measured as described with the following modifications^41^. Overnight exponential-phase cultures were subcultured into fresh PYE medium at OD_660_ = 0.02. Cultures were harvested by centrifugation after 6 hours (OD_660_ = 0.2–0.4) for exponential phase persister assays and after 24 hours (OD_660_ ~1.0) for stationary phase persister assays. Harvested cells were resuspended in fresh PYE medium for exponential phase persister assays or in spent medium (normalized by OD_660_) for stationary phase persister assays before aliquoting 1 ml/well into a 96 deep-well block. Antibiotic (streptomycin 25–150 μg/mL, kanamycin 25–150 μg/mL, or vancomycin 50–200 μg/mL) was added at a range of concentrations (10–500x MIC) to produce concentration-independent killing. The block was incubated with shaking, and at each time point, a sample was withdrawn and a ten-fold dilution series was performed in a 96-well plate using fresh PYE medium. These plates were allowed to regrow with incubation and shaking for 7 days. Regrowth in a well indicates that at least 1 surviving cell was present at the inoculation of that well. Survival was measured relative to the time zero CFU dilution plate. Survival was plotted against time to produce biphasic killing curves indicative of persistence. Reported data points are the mean and standard deviation of two biological replicates, each with six technical replicates. The persister frequency was estimated using the y-intercept of the curve describing the persister fraction.

### Assays of protein, RNA, and DNA synthesis

Overnight cultures were grown in PYE medium at 30 °C and subcultured in M2G minimal medium. M2G cultures were grown to OD_660_ ~0.2 before diluting back to OD_660_ = 0.02 in M2G supplemented with 1 mM theophylline and 1 mM IPTG to induce expression of HipA toxins. At the indicated times, 0.9 ml aliquots were withdrawn and added to 10 Ci of [^35^S]methionine (protein synthesis), 4 Ci [methyl-^3^H]thymidine (DNA synthesis), or 0.2 Ci [2-^14^C]uracil (RNA synthesis). After 2 min of incorporation, samples were chased for 10 min with 0.5 mg of methionine, 0.5 mg thymidine, or 0.5 mg uracil, respectively. Cells were harvested by centrifugation, resuspended in 200 µl cold 20% trichloroacetic acid (TCA), and further centrifuged at 15,000 × *g* for 30 min at 4 °C. Samples were washed twice with 1 ml cold 96% ethanol. Precipitates were transferred to vials, and the amount of incorporated radioactivity was counted in a liquid scintillation counter averaging counts per minute over 15 minutes using the appropriate energy setting for ^35^S or ^3^H isotopes. Samples were counted twice to verify counting accuracy.

### Phosphoproteome analysis

#### Bacterial growth, lysis and protein extraction

*C. crescentus* NA1000 grown in PYE medium (500 ml) were harvested at OD_600_ = 0.5 by centrifugation (20 min, 6,000 × *g*, 4 °C). The pellet was washed with 50 mM Tris-HCl, pH 8 and resuspended in 2.5 ml of this buffer. Halt Phosphatase Inhibitor cocktail (ThermoFisher #78420, 10 µl/ml sample) and Protease Inhibitor cocktail (Sigma #P8465, 1 μl/20 μl sample) were added just before cell disruption (1.6 kilobar). Debris was removed by centrifugation (10 min, 18,000 × *g*, 4 °C). Proteins were extracted with cold TCA/acetone (1:1 volume) at −20 °C for one hour. The sample was centrifuged (10 min, 18,000 × *g*, 4 °C) and washed with cold 10% β-mercaptoethanol in acetone. After centrifugation (20 min, 18,000 × *g*, 4 °C), the protein pellet was washed twice more as above. The pellet was washed once with cold methanol, centrifuged (20 min, 18,000 × *g*, 4 °C), dried, and solubilized in denaturing buffer (6 M urea, 2 M thiourea in 25 mM Tris-HCl, pH 8.0). Bradford assay revealed ~3 mg/ml protein.

#### Protein digestion

10 mg of proteins were reduced with 2 mM DTT and alkylated using 10 mM iodoacetamide in the dark. Proteins were digested with proteases at 1:100 w/w, first with Lys-C (Wako) for three hours at 30 °C, and after four-fold dilution with 20 mM ammonium bicarbonate, with sequencing grade modified trypsin (Promega) overnight at 30 °C.

#### Strong cation exchange (SCX) fractionation and phosphopeptide enrichment

SCX was performed on 10 mg of digested protein sample as described^56,57^. Phosphopeptides were enriched from SCX fractions using TiO_2_ beads as described^56,57^. For enrichment of phosphopeptides by immobilized metal affinity chromatography (IMAC), ten SCX fractions were dried and resuspended in 300 μl of 250 mM acetic acid, 30% acetonitrile (ACN) (v/v). Peptides were gently mixed with 80 μl of Phos-Select iron affinity gel (Sigma-Aldrich P9740) and incubated for 1.5 h using a tube rotator^58^. The mixture was transferred into SigmaPrep spin columns and washed twice with 200 μl of 250 mM acetic acid, 30% ACN (v/v), then once with 200 μl distilled water. Bound phosphopeptides were eluted with 100 μl 400 mM NH_4_OH, 30% ACN by centrifugation (1 min at 8,200 × *g*). Flow-through and elution fractions were dried almost completely (5–6 μl) under vacuum and stored at −20 °C until LC-MS/MS analysis.

#### Nano-liquid chromatography–tandem mass spectrometry

Mass spectrometry was performed on the PAPPSO platform (MICALIS Institute, INRAE, France, http://pappso.inra.fr/). A Q Exactive (Thermo Fisher Scientific) coupled to an Eksigent 2D nano LC (AB-Sciex, MA, USA) was used for nano-liquid chromatography–tandem mass spectrometry (LC-MS/MS) analysis. Samples were resuspended in a final volume of 12 μl 0.1% trifluoroacetic acid (TFA), 2% ACN, and 4 μl of sample were injected on the nano LC-Ultra system chain. The sample was loaded at 7.5 µl/min on the precolumn cartridge (C18, 5 µm, 120 Å, 20 mm, Nanoseparations) and desalted with 0.1% formic acid. Peptides were separated with a gradient of ACN on the reverse-phase column (stationary phase: C18 Biosphere, 3 µm; column: 75 µm i.d., 150 mm; Nanoseparations). Buffers were 0.1% formic acid in water (A) and 0.1% formic acid in ACN (B). Peptide separation was achieved with a linear gradient from 5% to 35% B for 28 min at 300 nl/min. Eluted peptides were analysed online using a nanoelectrospray interface. Ionisation (1.8 kV ionisation potential) was performed with stainless steel emitters (30 µm i.d.; Thermo Electron). Capillary temperature was 250 °C. Peptide ions were analysed with the following data-dependent acquisition steps: (i) full MS scan [mass-to-charge ratio (*m*/*z*) 400 to 1,400]; and (ii) MS/MS. Step 2 was repeated for the eight major ions detected in step 1. Dynamic exclusion was set to 40 s. The lock mass option “best” was chosen, MS resolution was 70,000 at *m*/*z* 400, auto gain control was 3e6 and maximum injection time was 250 ms. For MS2, the resolution was 17,500 at *m*/*z* 400, auto gain control was 2e5 with a maximum injection time of 120 ms, the isolation window was *m*/*z* = 3, the normalised collision energy was 30, the underfill ratio was 3%, the intensity threshold was 2.5e4, and the charge state was 2, 3.

#### Data processing and phosphopeptide validation

Identifications were performed using the *C. crescentus* NA1000 genome database (https://www.ncbi.nlm.nih.gov/nuccore/CP001340) supplemented by the contaminants database (trypsin, keratins, etc.). To increase confidence in phosphopeptide identification and add a significant number of phosphopeptides, two search engines were used in combination: MaxQuant (version 1.2.2.5, www.maxquant.org) and X!Tandem (version 2011.12.01.1,www.thegpm.org) with X!TandemPipeline v3.3.0, a bioinformatic tool developed by the PAPPSO platform (http://pappso.inra.fr/bioinfo/xtandempipeline/). The parameters for database searches were: one possible miscleavage, Cys carboxyamidomethylation set as a static modification, and Met oxidation and phosphorylation of tyrosine, serine and threonine residues set as variable modifications. Precursor mass and fragment mass tolerance were 10 ppm and 0.02 Da, respectively. Identified proteins were filtered and grouped using the X! TandemPipeline. Data filtering was achieved according to a peptide E value smaller than 0.05. The false discovery rate (FDR) at the peptide level was assessed from searches against a decoy database (using the reversed amino acid sequence for each protein). For MaxQuant, peptides composed of at least six amino acids were accepted, whereas those with a posterior error probability score >0.1 or an Andromeda score <70 were ignored. The maximum false discovery rate (FDR) at the protein and peptide levels as well as at the phosphorylation site level was set to 1%. All peptide sequences and phosphorylation sites showing a significant water regime effect were confirmed by manual inspection of the raw data to verify the peptide sequence and the phosphorylation site assignment.

## Supplementary information

Supplementary Dataset S1.

Supplementary Figures and Tables.

## Data Availability

All strains and plasmids used in this study can be obtained by contacting the corresponding author. The mass spectrometry proteomics data have been deposited to the ProteomeXchange Consortium via the PRIDE^59,60^ partner repository with Project Name: HipBA toxin-antitoxin system *Caulobacter crescentus*, and the dataset identifier PXD015525.
