# Supplementary material for: hipBA toxin-antitoxin systems mediate persistence in Caulobacter crescentus
Source: Sci Rep. 2020 Feb 18;10:2865. doi: 10.1038/s41598-020-59283-x (PMC7029023; doi:10.1038/s41598-020-59283-x)
Supplement: Supplementary file 2 — Supplementary Figures and Tables. [file 41598_2020_59283_MOESM2_ESM.pdf]

**Supplementary Information for**

# *hipBA* toxin-antitoxin systems mediate persistence in *Caulobacter crescentus*

**Charlie Y. Huang[1], Carlos Gonzalez-Lopez[1], Céline Henry[2], Ivan Mijakovic[3, 4], Kathleen R. Ryan[1]***

[1]Department of Plant & Microbial Biology, University of California, Berkeley, USA

[2]PAPPSO, Micalis Institute, Institut National de la Recherche Agronomique, AgroParisTech, Université Paris-Saclay, Jouy-en-Josas, France

[3]Division of Systems and Synthetic Biology, Department of Chemical and Biological Engineering, Chalmers University of Technology, Gothenburg, Sweden

[4]Novo Nordisk Foundation Center for Biosustainability, Technical University of Denmark, Kongens Lyngby, Denmark

This PDF includes Supplementary Figures S1-S10, Supplementary Tables S2-S4, and references cited in these elements.

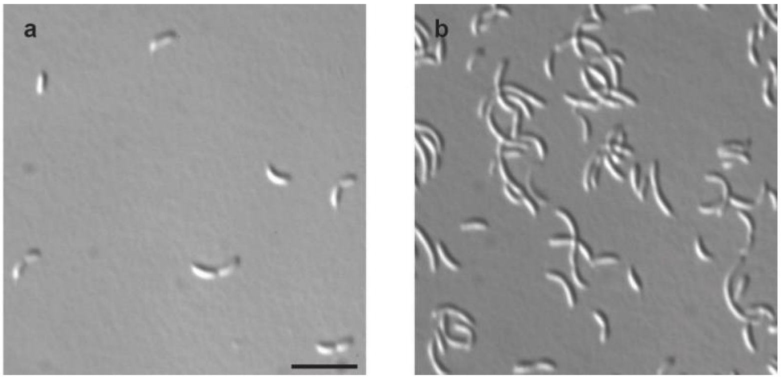


**Supplementary Figure S1. $HipA_3$ induction causes cell elongation. a**, NA1000 cells harboring p*FLAG-hipA₃* in exponential growth phase were diluted to $OD_{660}$ = 0.02 and split into two cultures. At time 0, $HipA_3$ expression was induced in one culture using IPTG and theophylline. Differential interference contrast micrographs were obtained after 9 h of growth at 30°C. **a**, Uninduced culture. **b**, $HipA_3$-expressing culture. Scale bar, 5 μm.

```
                                                                †
E.coli LIPEDETVTHPI----MA------WEKLTEARLEEVLTAYKADIPLGMIREENDFRISVA 152
```

```
E.coli ---MPKLVTWMN--NQRVGEL-TKLANGAHTFKYAPEWLASRYARPLSLSLPLQRGNITS 54
HipA1  MAMTRVLTVW--WGDGVVGSL-ALDETGDIRFAYDAAWLADEACPAVSVSLPKQPGAFSR 57
HipA2  MSGPAGLIVRMDGFNLPAGYL-ASDEARAISFAYDDRYIAAG-GPPLSLSMPLEQVSFGD 58
HipA3  MTTVAEVRLW---GS-RIGAVSLEDGAETAVFAYEPSFIASGI-QPAPLMMPLKAGVFSF 55

E.coli D--AVFNFF------DNLLPDSPI--VRDRIVKRY--HAKSRQPFDLLSEIGRDSVGAVT 102
HipA1  R--KTRSFF------AGLLPDDL---QRDNVARAL--GVSRQNDFGLLERLGGDVAGALT 104
HipA2  V--TARAFF------DNLLPEND---QMQRVMDRE--GLARDDIVGLLSHLGADCSGAIS 105
HipA3  PDLPPRSFHGLPGMLADALPDKYGHVLIDAWLATQGRSPESFNAVERLCYTGRRGMGALE 115
                                                                †
E.coli LIPEDETVTHPI----MA------WEKLTEARLEEVLTAYKADIPLGMIREENDFRISVA 152
HipA1  LWPEGEAPPKRQGGRTAE--------PLNDKRLLAILDELPR---RPFLAGEDGVRLSLA 153
HipA2  CLPIGADPIKVPGVLSED------YELLAPGAIAEIARSLAE---RQRLPDTITDPSPVA 156
HipA3  FSPMAGPRRRVSSKIDIDALVTLASEVLTHRH--DLRASFADADKADALRDILSVGTSAG 173

                                         #
E.coli GAQEKTALLRIGNDW--CIPK----GITPTTHIIKLPIGEIRQPNATLDLSQSVDNEYYC 206
HipA1  GAQEKLPVVLVDGAV--ALPA----LGQPSTHILKPANARWPA---------MTENEALA 198
HipA2  GVQRKIALTHTPQGFAKPRPG----RKVPTTHILKVPETRLRR---------DARLEAAA 203
HipA3  GARAKAVIAWNPATN-EVRSGQVEAGAGFGYWLLKFDGVSGNRDKELADPKGYGAVEHAY 232

E.coli LLLAKELGLNVPDAEIIKAGNVRALAVERFDRRWNAERTVLLRLPQEDMCQT----FGLP 262
HipA1  MRLAAAVGLPTAGVEPRRIGERTFLLVTRYDRVVSSD-GAVRRLHQEDFCQA----LGVS 253
HipA2  ARLASALGLDVSIPEAIVIDGVDALLITRFDRVV-RD-GVVYRLHQEDFAQA----MGLP 257
HipA3  GQMAAAAGIDVAESRLLEEGGRRHFMSKRFDRLDGGGK-----LHMQSLAAIAHLDFNDP 287

                                                         *    ‡ #
E.coli SSVKYESDGGPGIARIMA----FLMGSSEALKDRYDFMKFQVFQWLIGATDGHAKNFSVF 318
HipA1  PEHKYAAEGGPIFPDCF-NLVRNVCQPS--APAVLALLDAAIFNVIVGNADAHGKNYSLL 310
HipA2  ATLKYQRNGAPGRQFDAQAIARVLDQTEAPALSRTAFLSATIFNLLIGNTDNHAKNHGLL 317
HipA3  VANSYEQA-----LFTMR-----RMGLS--MAQLEEQFRRMVFNVLARNQDDHVKNIAFL 335

                    ‡
E.coli IQAGGSYRLTPFYDIISAFPVLGGTGIHISDLKLAMGLNASKGKKTAIDKIYPRHFLATA 378
HipA1  HQAG-AIVMAPLYDLMCTAAYPEVH------AKLAMKIAGR----AQLEAFKADTWRDFG 359
HipA2  YRQGRAPILAPLYDLLPSRMNLDFN------DQLSFNIGAA----DHPDAITFDDMMAFF 367
HipA3  MDRAGRWRLSPAFDITWSYNPDGEW---T--SRHQMSINGKR------DGFDFADLEACA 384

E.coli KVLRFPEVQMHEILSDFA---RMIPAALDNVKTSLPTDFPENVVT----AVESNVLRLHG 431
HipA1  RDIG------------------MGPAFVER-RANAQARRVLDQVGPVADAI--AACGFDR 398
HipA2  EVFG------------------MRRAAA--------ARFIENVIKPMIEALERATLGLAG 401
HipA3  KTASISRGHVGRIFDEVREAVMRWPTFADAA--GV-DERWRDQIG--------ATVRLEL 433

E.coli R-------------------------LSREYGSK-------- 440
HipA1  EALRGLRDLIAERARAVLK---LGQGER--------------- 423
HipA2  ERLKDMDDLIGRETEQLVEVLGLDVAVRERDYYPKVRHALAPS 444
HipA3  RR----------------------------------------- 435
```

**Supplementary Figure S2. Protein alignment of *E. coli* HipA and NA1000 HipA toxins**. Alignment was obtained using ClustalOmega. Functionally critical residues are indicated: *****, catalytic aspartate residue targeted for mutation; **‡**, $Mg^{++}$ binding residues; #, ATP-binding residues; **†**, autophosphorylated serine residue[1].

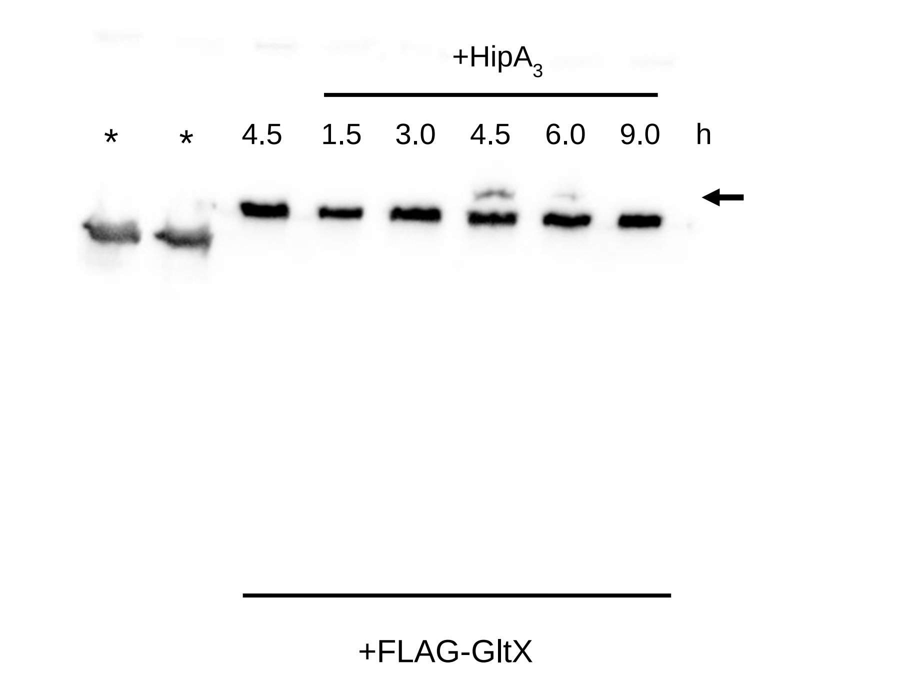


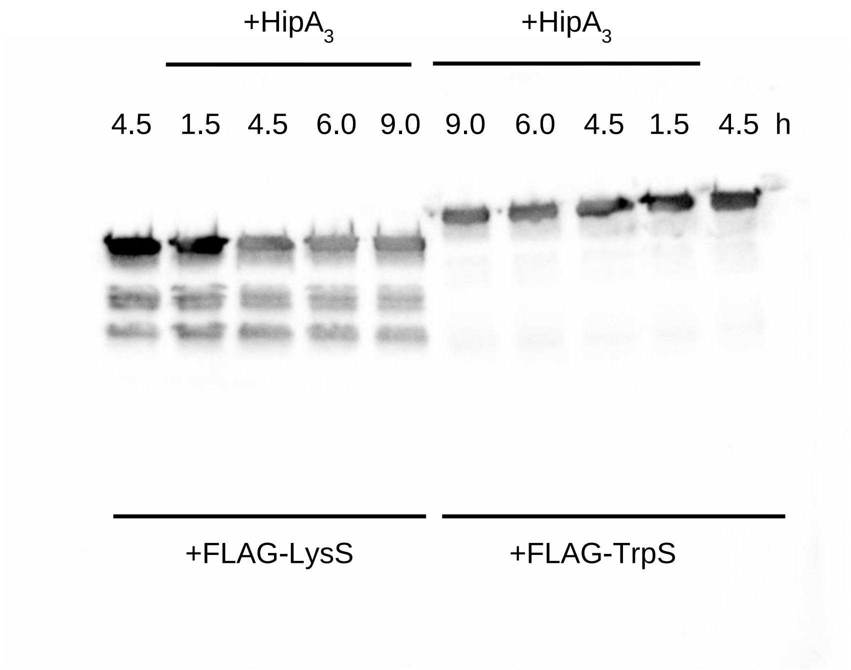


**Supplementary Figure S3**. HipA3 does not phosphorylate LysS or TrpS, and only weakly phosphorylates GltX. Full images are shown. *, protein samples not related to this experiment are shown in order to provide complete gel images. Arrow indicates phosphorylated FLAG-GltX species with reduced mobility in *Caulobacter* coexpressing $HipA_3$. Molecular size standards are omitted because they do not migrate at the correct sizes in Phos-tag gels, according to manufacturer's information (APExBIO).

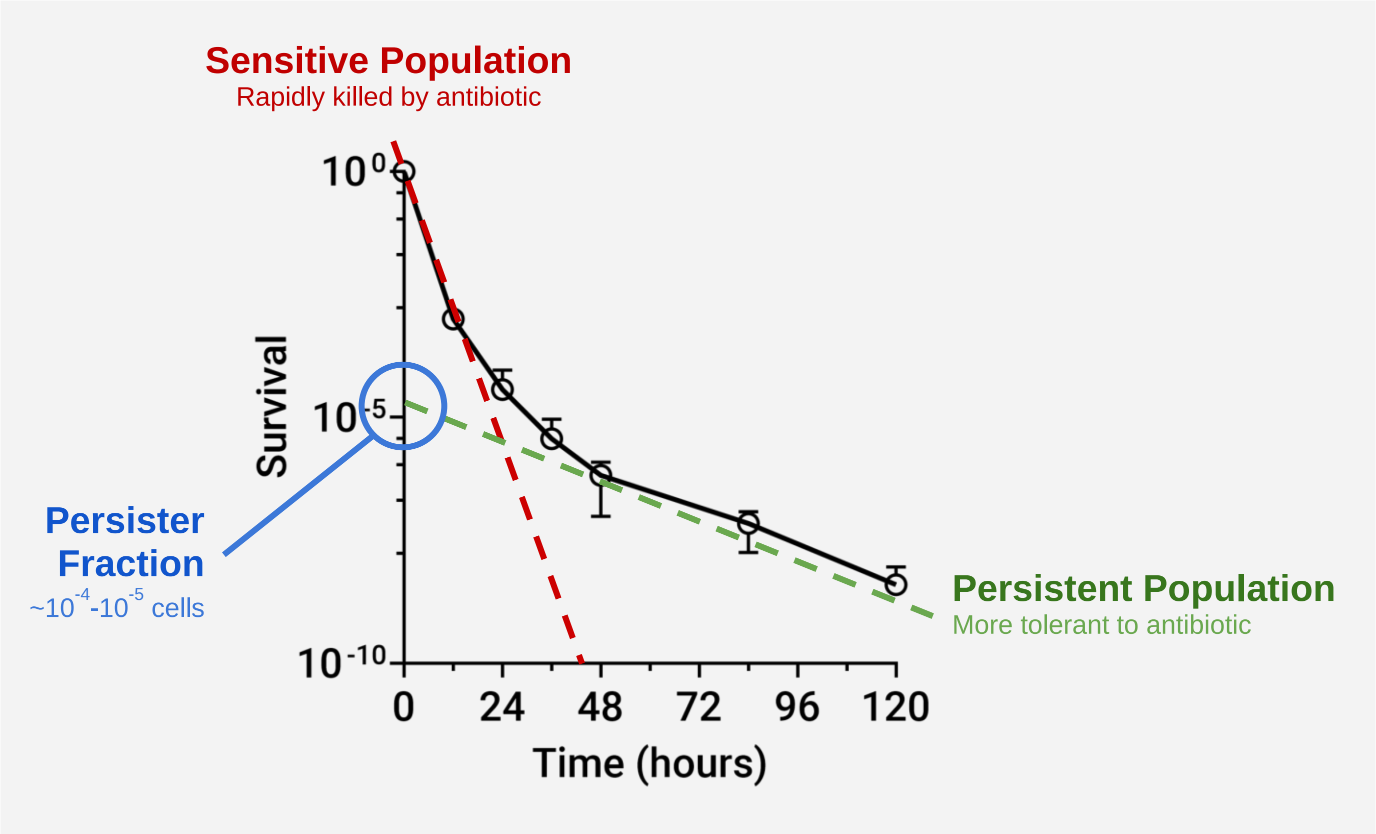


**Supplementary Figure S4. Biphasic killing curves describe two sub-populations (sensitive and persistent) and their different rates of killing by antibiotics.** The first phase primarily describes the survival of the sensitive fraction, while the second phase describes the killing of the remaining persistent population. The persister fraction can be approximated using the y-intercept of the second phase of the curve. This explanatory diagram was created using actual experimental data that re-appears in Supplementary Fig. S5.

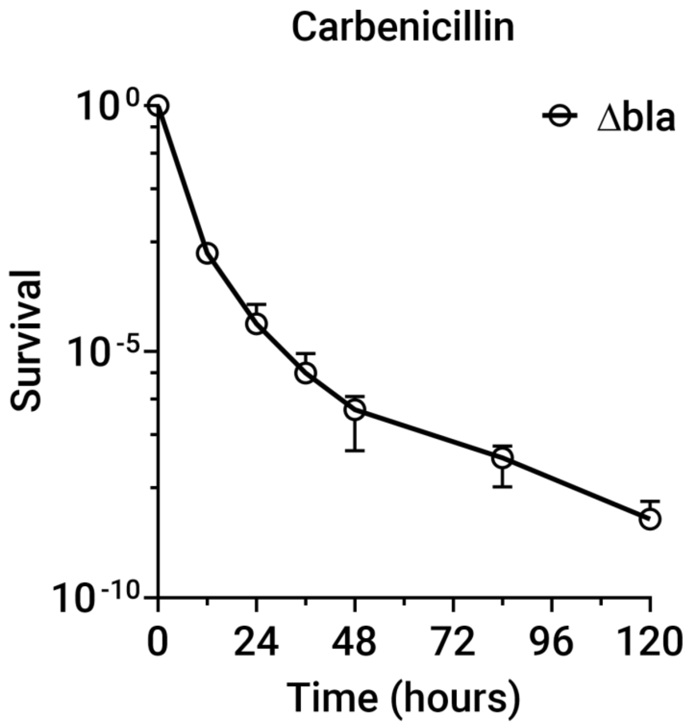


**Supplementary Figure S5. The *C. crescentus* NA1000 *Δbla* strain displays persistence toward carbenicillin.** The *Δbla* strain was grown to stationary phase in PYE and analyzed in a persistence assay using 100 µg/ml carbenicillin as described in Methods. The MIC of carbenicillin toward NA1000 *Δbla* was measured at 10-20 µg/ml.

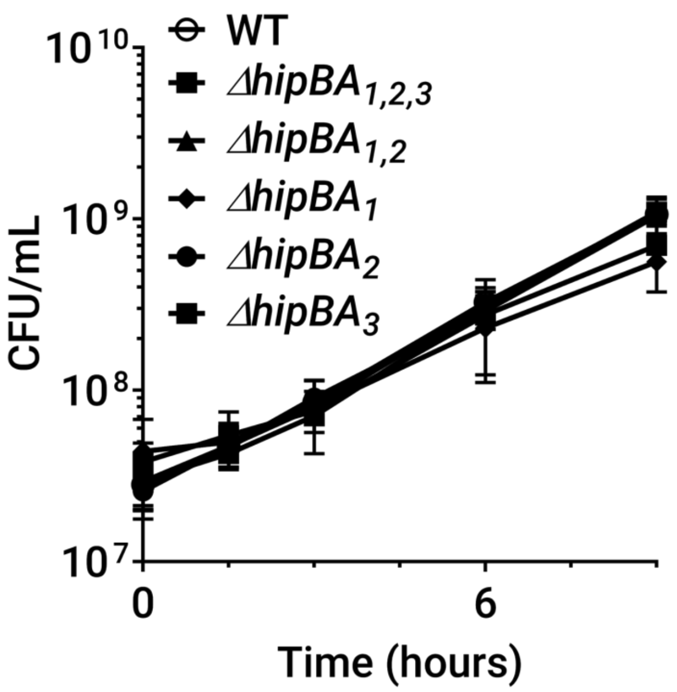


**Supplementary Figure S6. NA1000 *ΔhipBA* strains do not have a growth defect.** Growth curves of NA1000 and the Δ*hipBA* operon knockout strains in PYE medium. Exponential phase cultures were diluted to $OD_{660}$= 0.02 at time zero. At the indicated times, samples were withdrawn for CFU enumeration. Data points are the mean and standard deviation of three independent biological replicates.

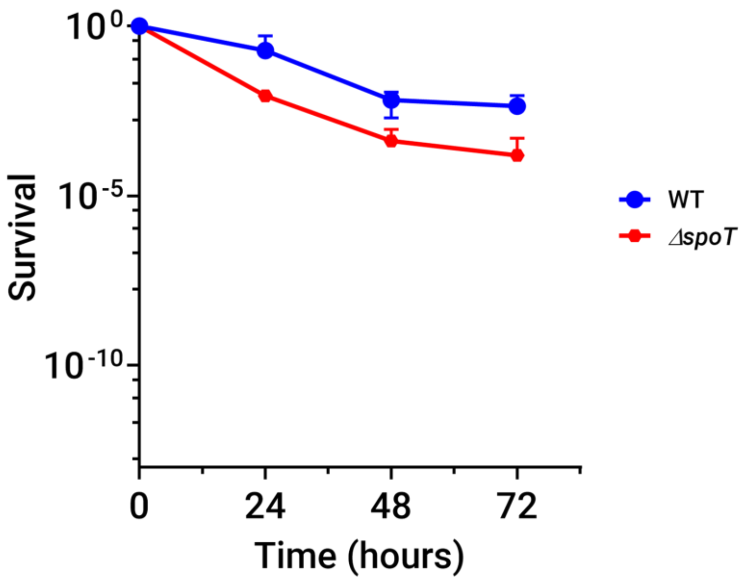


**Supplementary Figure S7. The *ΔspoT* strain has reduced tolerance to the conditions of stationary phase in PYE.** Exponential phase cultures of WT and *ΔspoT* strains were diluted to $OD_{660}$= 0.02 and grown to stationary phase in PYE medium. Samples were withdrawn for CFU enumeration at the indicated times. Data points are the means of two independent biological replicates, each with six technical replicates.

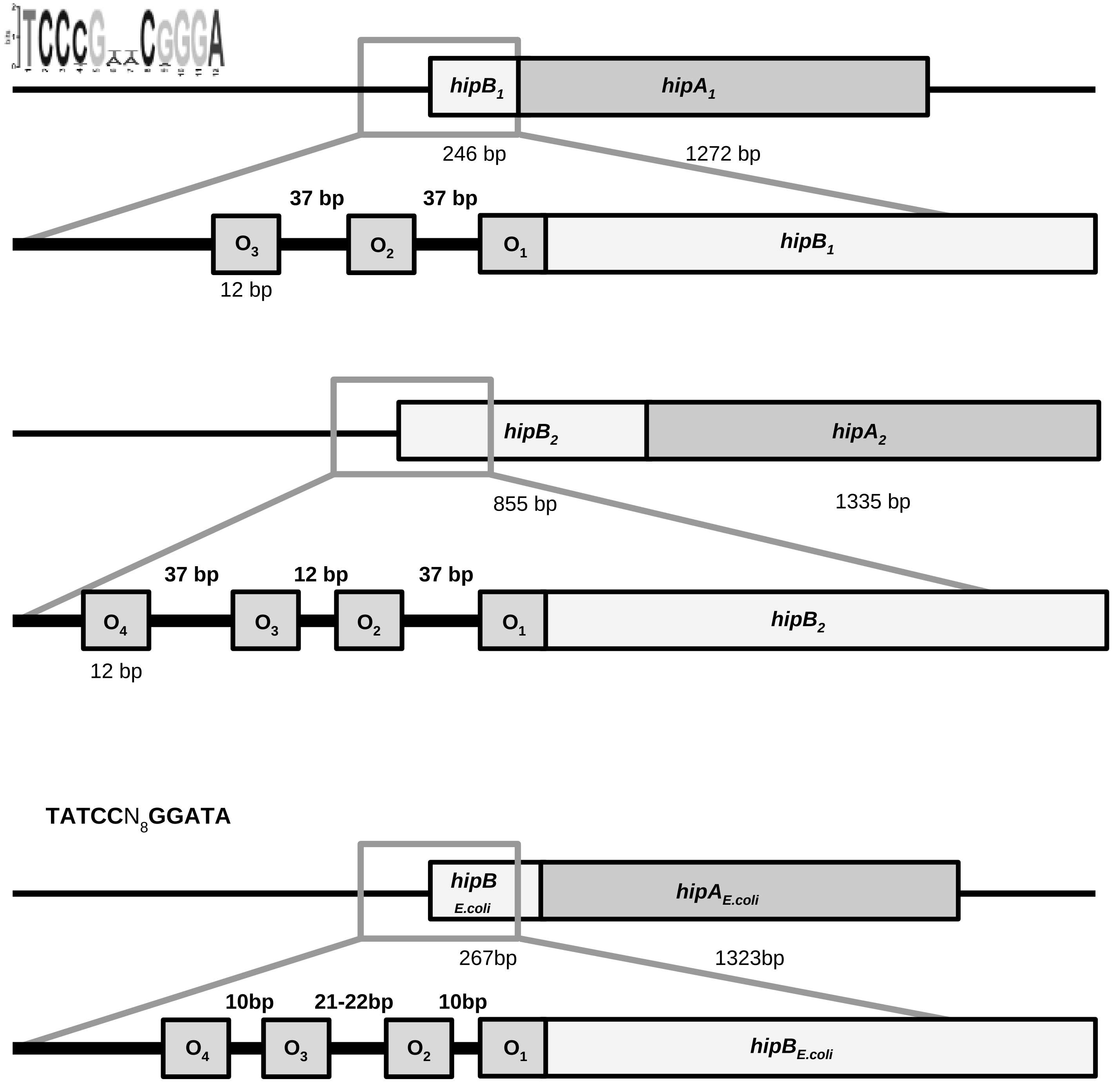


**Supplementary Figure S8. Identical putative binding motifs with the indicated spacing are observed in the promoter regions upstream of *hipBA₁* and *hipBA₂*.** Repetitive inverted repeats were identified using the EMBOSS bioinformatics suite[2]. The *E. coli hipBA* operon and HipB-binding operator sequence are included for comparison.

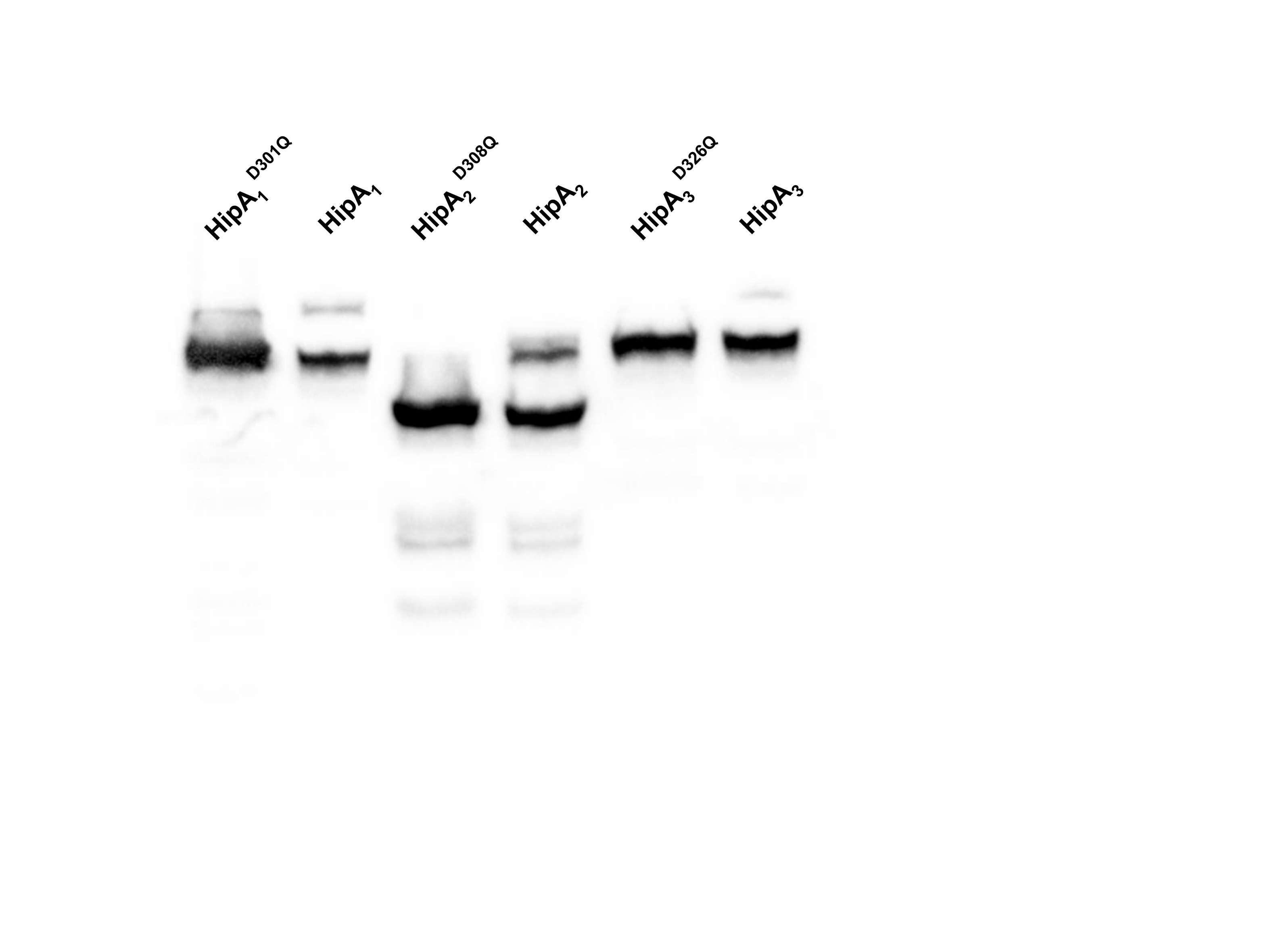


**Supplementary Figure S9.** Full gel image corresponding to Fig. 1d. Whole-cell lysates were analyzed by Phos-tag mobility shift and Western blotting with anti-FLAG antibodies. Molecular size standards are omitted because they do not migrate at the correct sizes in Phos-tag gels, according to manufacturer's information (APExBIO).

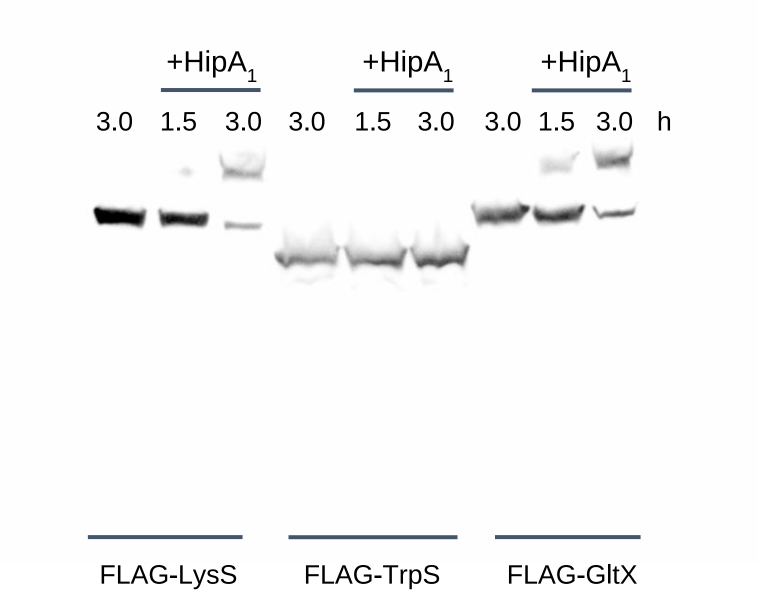


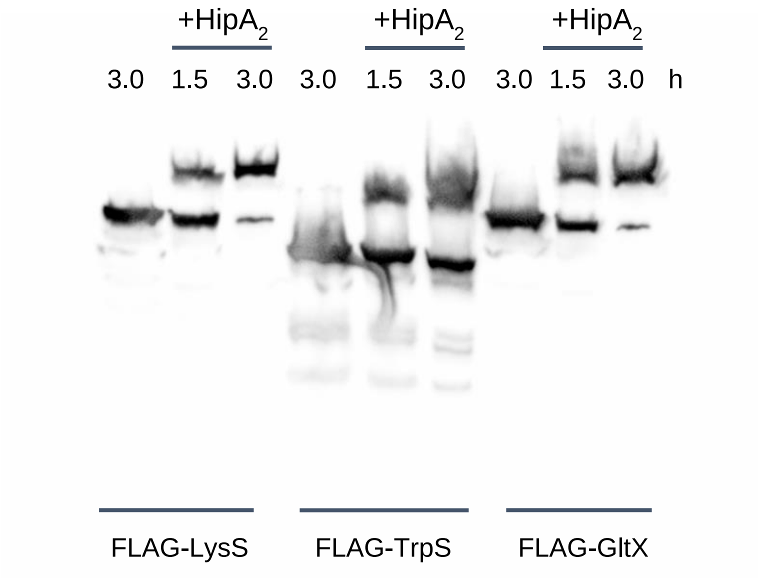


**Supplementary Figure S10.** Full gel images corresponding to Fig. 4. Molecular size standards are omitted because they do not migrate at the correct sizes in Phos-tag gels, according to manufacturer's information (APExBIO).

**Supplementary Table S2. Strains used in this study.**

| Number | Description | Expanded genotype | Reference |
|---|---|---|---|
| KR3707 | NA1000 or wild-type (WT) | *Caulobacter crescentus* NA1000 | [3] |
| KR4280 | WT + pFLAG-*hipA*$_1$ | | This study |
| KR4281 | WT + pFLAG-*hipA*$_2$ | | This study |
| KR4282 | WT + pFLAG-*hipA*$_3$ | | This study |
| KR658 | *Δbla* | | [4] |
| KR3744 | *ΔhipBA*$_1$ | | This study |
| KR3714 | *ΔhipBA*$_2$ | | This study |
| KR3631 | *ΔhipBA*$_3$ | | This study |
| KR3794 | *ΔhipBA*$_{1,2}$ | *ΔhipBA*$_1$ *ΔhipBA*$_2$ | This study |
| KR3750 | *ΔhipBA*$_{1,2,3}$ | *ΔhipBA*$_1$ *ΔhipBA*$_2$ *ΔhipBA*$_3$ | This study |
| KR4283 | *ΔhipBA*$_{1,2,3}$ + pMR20 | | This study |
| KR4284 | *ΔhipBA*$_{1,2,3}$ + pJS71 + pMR20 | | This study |
| KR4285 | *ΔhipBA*$_{1,2,3}$ + pFLAG-*hipA*$_1$ | | This study |
| KR4286 | *ΔhipBA*$_{1,2,3}$ + pFLAG-*hipA*$_2$ | | This study |
| KR4287 | *ΔhipBA*$_{1,2,3}$ + pFLAG-*hipA*$_3$ | | This study |
| KR4288 | *ΔhipBA*$_{1,2,3}$ + pFLAG-*hipA*$_1$ + pJS71 | | This study |
| KR4289 | *ΔhipBA*$_{1,2,3}$ + pFLAG-*hipA*$_2$ + pJS71 | | This study |
| KR4290 | *ΔhipBA*$_{1,2,3}$ + pFLAG-*hipA*$_3$ + pJS71 | | This study |
| KR4291 | *ΔhipBA*$_{1,2,3}$ + pFLAG-*hipA*$_1$D301Q | | This study |
| KR4292 | *ΔhipBA*$_{1,2,3}$ + pFLAG-*hipA*$_2$D308Q | | This study |
| KR4293 | *ΔhipBA*$_{1,2,3}$ + pFLAG-*hipA*$_3$D326Q | | This study |
| KR4294 | *ΔhipBA*$_{1,2,3}$ + pMR20 + pFLAG-*hipB*$_1$ | | This study |
| KR4295 | *ΔhipBA*$_{1,2,3}$ + pMR20 + pFLAG-*hipB*$_2$ | | This study |
| KR4296 | *ΔhipBA*$_{1,2,3}$ + pMR20 + pFLAG-*hipB*$_3$ | | This study |
| KR4297 | *ΔhipBA*$_{1,2,3}$ + pFLAG-*hipA*$_1$ + pFLAG-*hipB*$_1$ | | This study |
| KR4298 | *ΔhipBA*$_{1,2,3}$ + pFLAG-*hipA*$_2$ + pFLAG-*hipB*$_1$ | | This study |
| KR4299 | *ΔhipBA*$_{1,2,3}$ + pFLAG-*hipA*$_3$ + pFLAG-*hipB*$_1$ | | This study |
| KR4300 | *ΔhipBA*$_{1,2,3}$ + pFLAG-*hipA*$_1$ + pFLAG-*hipB*$_2$ | | This study |

| | | | |
|---|---|---|---|
| KR4301 | $\Delta hipBA_{1,2,3}$+ pFLAG-$hipA_2$ + pFLAG-$hipB_2$ | | This study |
| KR4302 | $\Delta hipBA_{1,2,3}$ + pFLAG-$hipA_3$ + pFLAG-$hipB_2$ | | This study |
| KR4303 | $\Delta hipBA_{1,2,3}$ + pFLAG-$hipA_1$ + pFLAG-$hipB_3$ | | This study |
| KR4304 | $\Delta hipBA_{1,2,3}$ + pFLAG-$hipA_2$ + pFLAG-$hipB_3$ | | This study |
| KR4305 | $\Delta hipBA_{1,2,3}$ + pFLAG-$hipA_3$ + pFLAG-$hipB_3$ | | This study |
| KR3063 | *ΔspoT* | | [5] |
| KR4306 | *ΔspoT* + pMR20 | | This study |
| KR4307 | *ΔspoT* + pFLAG-$hipA_1$ | | This study |
| KR4308 | *ΔspoT* + pFLAG-$hipA_2$ | | This study |
| KR4309 | *ΔspoT* + pFLAG-$hipA_3$ | | This study |
| KR4310 | WT + pFLAG-*metG* | | This study |
| KR4311 | WT + pFLAG-*lysS* | | This study |
| KR4312 | WT + pFLAG-*trpS* | | This study |
| KR4313 | WT + pFLAG-*gltX* | | This study |
| KR4314 | WT + pMR20 + pFLAG-*lysS* | | This study |
| KR4315 | WT + pMR20 + pFLAG-*trpS* | | This study |
| KR4316 | WT + pMR20 + pFLAG-*gltX* | | This study |
| KR4317 | WT + p$hipA_1$ + pFLAG-*lysS* | | This study |
| KR4318 | WT + p$hipA_1$ + pFLAG-*trpS* | | This study |
| KR4319 | WT + p$hipA_1$ + pFLAG-*gltX* | | This study |
| KR4320 | WT + p$hipA_2$ + pFLAG-*lysS* | | This study |
| KR4321 | WT + p$hipA_2$ + pFLAG-*trpS* | | This study |
| KR4322 | WT + p$hipA_2$ + pFLAG-*gltX* | | This study |
| KR4323 | WT + p$hipA_3$ + pFLAG-*lysS* | | This study |
| KR4324 | WT + p$hipA_3$ + pFLAG-*trpS* | | This study |
| KR4325 | WT + p$hipA_3$ + pFLAG-*gltX* | | This study |

**Supplementary Table S3. Plasmids used in this study.**

| Name | Description | Reference |
|---|---|---|
| pMR20 | Broad-host-range, low-copy-number vector; *tetAR* | [6] |
| pJS71 | Broad host-range cloning vector; high copy; chlor$^R$; pBBR1MCS derivative with unique EcoRI site | [7] |
| pNPTS138 | kan$^R$; *sacB*-containing integration vector | GenBank Accession Number MK533795.1 |
| pCYH1 | pNPTS138-*hipBA$_1$*KO (*CCNA_00481-00482*) | This study |
| pCYH2 | pNPTS138-*hipBA$_2$*KO (*CCNA_02821-02822*) | This study |
| pCYH3 | pNPTS138-*hipBA$_3$*KO (*CCNA_02858-02859*) | This study |
| p*hipA$_1$* | pMR20 - PriboA - *hipA$_1$*(*CCNA_00482*) | This study |
| p*hipA$_2$* | pMR20 - PriboA - *hipA$_2$*(*CCNA_02821*) | This study |
| p*hipA$_3$* | pMR20 - PriboA - *hipA$_3$*(*CCNA_02858*) | This study |
| pFLAG-*hipA$_1$* | pMR20 - PriboA - FLAG-*hipA$_1$* | This study |
| pFLAG-*hipA$_2$* | pMR20 - PriboA - FLAG-*hipA$_2$* | This study |
| pFLAG-*hipA$_3$* | pMR20 - PriboA - FLAG-*hipA$_3$* | This study |
| pFLAG-*hipA$_1$*$^{D301Q}$ | pMR20 - PriboA - FLAG-*hipA$_1$*D301Q | This study |
| pFLAG-*hipA$_2$*$^{D308Q}$ | pMR20 - PriboA - FLAG-*hipA$_2$*D308Q | This study |
| pFLAG-*hipA$_3$*$^{D326Q}$ | pMR20 - PriboA -pFLAG-*hipA$_3$*D326Q | This study |
| pFLAG-*hipB$_1$* | JS71 - PriboA - FLAG-*hipB$_1$*(*CCNA_00481*) | This study |
| pFLAG-*hipB$_2$* | JS71 - PriboA - FLAG-*hipB$_2$*(*CCNA_02822*) | This study |
| pFLAG-*hipB$_3$* | JS71 - PriboA - FLAG-*hipB$_3$*(*CCNA_02859*) | This study |
| pFLAG-*metG* | JS71 - PriboA - FLAG-*metG*(*CCNA_01547*) | This study |
| pFLAG-*lysS* | JS71 - PriboA - FLAG-*lysS*(*CCNA_00082*) | This study |
| pFLAG-*trpS* | JS71 - PriboA - FLAG-*trpS*(*CCNA_00062*) | This study |
| pFLAG-*gltX* | JS71 - PriboA - FLAG-*gltX* (*CCNA_01982*) | This study |
| p*hipBA$_1$* | pMR20 - *hipBA$_1$* | This study |
| p*hipBA$_2$* | pMR20 - *hipBA$_2$* | This study |
| p*hipBA$_3$* | pMR20 - *hipBA$_3$* | This study |
| p*hipBA$_{1,2}$* | pMR20 - *hipBA$_{1,2}$* | This study |

**Supplementary Table S4. Primers used in this study.**

| Name | Sequence (5'-3') | Purpose |
|---|---|---|
| hipBA1 Up F | attgaagccggctggcgccaTCGACCCGGGCGATCTTC | *hipBA₁* chromosomal knockout |
| hipBA1 Up R | tctagcgctcGAGGGTCATGAAAATTCTCCCG | |
| hipBA1 Dn F | catgaccctcGAGCGCTAGACTCGATCCCGC | |
| hipBA1 Dn R | cgtcacggccgaagctagcgGCCCAGGACGGCGACACC | |
| hipBA2 Up F | attgaagccggctggcgccaTTGCCTCTTGAGGAGCCG | *hipBA₂* chromosomal knockout |
| hipBA2 Up R | acacctcctaCAGGAGCATAAGTAGCTAACGAAG | |
| hipBA2 Dn F | tatgctcctgtagGAGGTGTTCGGCATGAGG | |
| hipBA2 Dn R | cgtcacggccgaagctagcgAAGATCACCCCGCTGTCG | |
| hipBA3 Up F | attgaagccggctggcgccaCCCGGGCGACAAGAAGGC | *hipBA₃* chromosomal knockout |
| hipBA3 Up R | cctaccgcctAAATTTCATAAATCGCCTCTTGTGGCG | |
| hipBA3 Dn F | tatgaaatttAGGCGGTAGGCGGGGGAT | |
| hipBA3 Dn R | cgtcacggccgaagctagcgGACAGCGGCACCAGCCGTC | |
| riboA to pJS71 F | cggccgctctagaactagtgTGGTGCAAAACCTTTCGC | pJS71 protein expression vector |
| riboA to pMR10 or pMR20 F | ctatgaccatgattacgccaTGGTGCAAAACCTTTCGC | pMR10/pMR20 expression vector |
| riboA to FLAG R | cttgtcgtcatcgtctttgtagtcCATCTTGTTGATACCCCC | riboA promoter for protein expression |
| riboA to hipA1 R | tagtcatcgcCATCTTGTTGATACCCCC | |
| riboA to hipA2 R | cgggcccactCATCTTGTTGATACCCCC | |
| riboA to hipA3 R | cgacggtggtCATCTTGTTGATACCCCC | |
| hipA1 to riboA F | caacaagatgGCGATGACTAGAGTGCTGACG | *hipA₁* for pMR20 expression vector |
| hipA1 F | gactacaaagacgatgacgacaagGCGATGACTAGAGTGCTGACG | FLAG-hipA1 for pMR20 expression vector |
| hipA1 R | ttgtaaaacgacggccagtgCTAGCGCTCGCCCTGACC | |
| hipA2 to riboA F | caacaagatgAGTGGGCCCGCAGGCCTG | *hipA₂* for pMR20 expression vector |
| hipA2 F | gactacaaagacgatgacgacaagAGTGGGCCCGCAGGCCTG | FLAG-*hipA₂* for pMR20 expression vector |
| hipA2 R | ttgtaaaacgacggccagtgTCAACTCGGCGCCAAGGCG | |
| hipA3 to riboA F | caacaagatgACCACCGTCGCCGAGGTT | *hipA₃* for pMR20 expression vector |
| hipA3 F | gactacaaagacgatgacgacaagACCACCGTCGCCGAGGTT | FLAG-*hipA₃* for pMR20 expression vector |
| hipA3 R | ttgtaaaacgacggccagtgCTACCGCCTGAGCTCCAG | |
| hipA1 D301Q F | tcttgccgtgagcctgggcgttgccgacg | *hipA₁* D301Q mutagenic primers |
| hipA1 D301Q R | cgtcggcaacgcccaggctcacggcaaga | |
| hipA2 D308Q F | tggttcttggcgtggttctgagtattgccaatcagaagatt | *hipA₂* D308Q mutagenic primers |
| hipA2 D308Q R | aatcttctgattggcaatactcagaaccacgccaagaacca | |
| hipA3 D326Q F | gttcttgacgtggtcctgctggtttcgcgccag | *hipA₃* D326Q mutagenic primers |
| hipA3 D326Q R | ctggcgcgaaaccagcaggaccacgtcaagaac | |
| hipB1 F | gactacaaagacgatgacgacaagACCCTCCTCCGGCAAGGC | FLAG-*hipB₁* for pJS71 expression vector |
| hipB1 R | cgaggtcgacggtatcgataCTAGTCATCGCCACCGCTCAC | |
| hipB2 F | gactacaaagacgatgacgacaagCTCCTGAGCGGGAGCGAAAAC | FLAG-*hipB₂* for pJS71 expression vector |
| hipB2 R | cgaggtcgacggtatcgataTCATCTGGGCGCCGCCAT | |
| hipB3 F | gactacaaagacgatgacgacaagAAATTTGAATCTCTCCTGTCC | FLAG-*hipB₃* for pJS71 expression vector |
| hipB3 R | cgaggtcgacggtatcgataTCATTTTTCATCGCCCCAC | |

| | | |
|---|---|---|
| gltX F | gactacaaagacgatgacgacaagTCGAACCCCACCCCTACC | FLAG-*gltX* for pJS71 expression vector |
| gltX R | cgaggtcgacggtatcgataTCATGCGCGAGGCGCTAG | |
| lysS F | gactacaaagacgatgacgacaagTTTGAAGGCCTCTCCCCC | FLAG-*lysS* for pJS71 expression vector |
| lysS R | cgaggtcgacggtatcgataTCAGCCCAGCTTTTCCTC | |
| metG F | gactacaaagacgatgacgacaagGCTCGCATCCTGATTACCTCCG | FLAG-*metG* for pJS71 expression vector |
| metG R | cgaggtcgacggtatcgataTTACTCCGCGCCGCCGAA | |
| trpS F | gactacaaagacgatgacgacaagACCGACCAAGCTCCCGTC | FLAG-*trpS* for pJS71 expressi2n vector |
| trpS R | cgaggtcgacggtatcgataCTACGCGCCCCAGAAGCC | |
| hipBA1 F | ctatgaccatgattacgccaGGGGCCCAGCCCCATGGC | *hipBA*$_1$ operon and promoter for pMR10 complementation vector |
| hipBA1 R | ttgtaaaacgacggccagtgCTAGCGCTCGCCCTGACCCAG | |
| hipBA2 F | ctatgaccatgattacgccaTCAACTCGGCGCCAAGGC | *hipBA*$_2$ operon and promoter for pMR10 complementation vector |
| hipBA2 R | ttgtaaaacgacggccagtgCTGCGGGGAGGGTGTGGG | |
| hipBA1 to hipBA2 R | gccgagttgaCTAGCGCTCGCCCTGACCCAG | *hipBA*$_1$ and *hipBA*$_2$ operons for pMR10 complementation vector |
| hipBA2 to hipBA1 F | cgagcgctagTCAACTCGGCGCCAAGGC | |
| CCNA_03445 qPCR F | CTGGATCTGTCTCGCCTTGG | RT-PCR oligonucleotides |
| CCNA_03445 qPCR R | GGTGGGATGGGGCTTCTG | |
| CCNA_03181 qPCR F | TTCAGACGCTTGAGACCACC | |
| CCNA_03181 qPCR R | CGTACATGGTTTTCGGCACG | |
| CCNA_01674 qPCR F | CAGACCCTAGCGGACACTCC | |
| CCNA_01674 qPCR R | GGCGTGAACATCTCGTCGTA | |
| CCNA_01596 qPCR F | ATGTTTCCCGACACCGACTC | |
| CCNA_01596 qPCR R | CTGCTCGGGGTTCAGGC | |
| CCNA_00520 qPCR F | GCGATCGCGGAATATCTCGAC | |
| CCNA_00520 qPCR R | CTGGTCGACTGGATGACGAA | |
| rho1 F | AGACACCGAAAACCAGGTTCC | |
| rho1 R | CGCTTCAGTGGTGATGTCGG | |
| rho2 F | ACCGAAGACACCGAAAACCAG | |
| rho2 R | GCCGCTTCAGTGGTGATGT | |

**References**

1. Correia, F. F. *et al*. Kinase activity of overexpressed HipA is required for growth arrest and multidrug tolerance in *Escherichia coli*. *J.Bacteriol.* **188**, 8360-8367 (2006).

2. Rice, P., Longden, I. & Bleasby, A. EMBOSS: The European Molecular Biology Open Software Suite. *Trends Genet.* **16**, 276-277 (2000).

3. Evinger, M. & Agabian, N. Envelope-associated nucleoid from *Caulobacter crescentus* stalked and swarmer cells. *J.Bacteriol.* **132**, 294-301 (1977).

4. Docquier, J. D. *et al*. CAU-1, a subclass B3 metallo-beta-lactamase of low substrate affinity encoded by an ortholog present in the *Caulobacter crescentus* chromosome. *Antimicrob. Agents Chemother.* **46**, 1823-1830 (2002).

5. Boutte, C. C. & Crosson, S. The complex logic of stringent response regulation in *Caulobacter crescentus*: starvation signalling in an oligotrophic environment. *Mol.Microbiol.* **80**, 695-714 (2011).

6. Roberts, R. C. *et al*. Identification of a *Caulobacter crescentus* operon encoding *hcrA*, involved in negatively regulating heat-inducible transcription, and the chaperone gene *grpE*. *J.Bacteriol.* **178**, 1829-1841 (1996).

7. Jensen, R. B., Wang, S. C. & Shapiro, L. A moving DNA replication factory in *Caulobacter crescentus*. *EMBO J.* **20**, 4952-4963 (2001).
